# Iron mobilization during lactation reduces oxygen stores in a diving mammal

**DOI:** 10.1038/s41467-022-31863-7

**Published:** 2022-08-02

**Authors:** Michelle R. Shero, Amy L. Kirkham, Daniel P. Costa, Jennifer M. Burns

**Affiliations:** 1 Biology Department, Woods Hole Oceanographic Institution, 266 Woods Hole Rd, Woods Hole, MA 02543 US; 2 Department of Biological Sciences, University of Alaska Anchorage, 3101 Science Circle, Anchorage, AK 99508 USA; 3 College of Fisheries and Ocean Sciences, University of Alaska Fairbanks, 17101 Point Lena Loop Road, Juneau, AK 99801 USA; 4 Institute of Marine Science, Ecology and Evolutionary Biology, University of California Santa Cruz, Santa Cruz, CA 95060 US; 5 Department of Biological Sciences, Texas Tech University, Lubbock, TX 79409 USA

**Keywords:** Ecophysiology, Homeostasis, Animal physiology

## Abstract

The profound impacts that maternal provisioning of finite energy resources has on offspring survival have been extensively studied across mammals. This study shows that in addition to calories, high hemoprotein concentrations in diving mammals necessitates exceptional female-to-pup iron transfer. Numerous indices of iron mobilization (ferritin, serum iron, total-iron-binding-capacity, transferrin saturation) were significantly elevated during lactation in adult female Weddell seals (*Leptonychotes weddellii*), but not in skip-breeders. Iron was mobilized from endogenous stores for incorporation into the Weddell seal’s milk at concentrations up to 100× higher than terrestrial mammals. Such high rates of iron offload to offspring drew from the female’s own heme stores and led to compromised physiologic dive capacities (hemoglobin, myoglobin, and total body oxygen stores) after weaning their pups, which was further reflected in shorter dive durations. We demonstrate that lactational iron transfer shapes physiologic dive thresholds, identifying a cost of reproduction to a marine mammal.

## Abstract (web summary)

Here, the authors show that Weddell seal mothers mobilize endogenous iron stores during lactation to provide to pups, resulting in iron concentrations in milk 100x higher than terrestrial mammals. This was associated with reduced dive durations in the mother, a cost of reproduction.

## Introduction

Balancing allocation of finite resources during reproduction has important implications for future survival of both female and offspring^1^. Across mammals, lactation is the most energetically-costly aspect of reproduction^2^ and the caloric demands of offspring-rearing are met using various life history strategies, ranging from income to capital breeding^3^. Income breeders rely on resource availability concurrent with lactation, whereas capital breeding females accrue resources across gestation and this stored energetic ‘capital’ supports most (or all) of the costs of milk provisioning while females remain temporally and spatially separated from prey resources^3–7^. Patterns of profound female-to-offspring mass and caloric transfer have been extensively studied in mammalian systems^3–7^. In addition to calories, females also transfer micronutrients, hormones, and other bioactive compounds to offspring through their milk^8–10^. However, much less effort has been made to understand factors determining how these compounds are parsed between offspring development versus the female’s own self-maintenance, and the potential long-term maternal costs that transfer and depletion of micronutrients may have.

Diving mammals may provide a particularly tractable system for study of the maternal-offspring ‘tug-of-war’ over micronutrients due to the high iron demands necessary to maintain high tissue hemoprotein levels with which to carry oxygen (O_2_)^11–13^. Large endogenous O_2_ stores dictate how proficiently marine mammals can forage underwater, and result from the evolution of large blood volumes (to 22% body mass in marine mammals, compared to 7% in terrestrial mammals), elevated hematocrit (Hct, to 65% vs. 40–50% in terrestrial mammals), high hemoglobin concentrations (Hb, to 24 g·dL^−1^ vs 12–17 g·dL^−1^) within red blood cells, and large muscle masses with exceptionally high myoglobin concentrations (Mb, to 120 mg·g^−1^ vs. 5–10 mg·g^−1^)^12,13^. In combination, marine mammals have 2.5–5× higher mass-specific total body oxygen (TBO_2_) stores compared with terrestrial mammals, and species that dive longer and deeper have greater hemoprotein concentrations and O_2_ stores^12^. Marine mammals also have a suite of adaptations for slow and efficient use of on-board O_2_ stores to prolong dive durations. Diving metabolic rates (DMR) are suppressed through bradycardia, selective vasoconstriction prioritizing O_2_ delivery to anoxia-intolerant tissues, and lowering body temperature^13,14^. Locomotor activities are further supported by elevated muscle catabolic enzyme activities and high mitochondrial and lipid droplet densities^15–19^. Large O_2_ stores paired with slow O_2_ consumption allows marine mammals to extend dive durations while relying on aerobic metabolism (i.e., staying below the aerobic dive limit, ADL, prior to a rise in lactate production). In nature, the majority of marine mammal dives remain aerobic, and thus the ADL represents an important ecological threshold that places physiological constraints on foraging success^20^.

An animal’s foraging success and prey intake provide a source of iron, and gastrointestinal absorption of iron is tightly regulated to maintain homeostasis because mammals lack a means of iron excretion^21^. Dietary iron uptake is regulated by the gut enterocytes, where iron undergoes multiple redox reactions during absorption. Once divalent metal transporter (DMT-1) shuttles dietary iron into the intestinal epithelial cell, iron is exported across the basolateral membrane by ferroportin (FPN1) and transported through circulation bound to transferrin (Tf) glycoproteins^21^. Without sufficient Tf to bind and chelate iron, free iron would otherwise lead to the generation of reactive oxygen species (ROS), cellular damage, and organ dysfunction^21^. The Tf-iron complex has receptors on most body cells to facilitate iron delivery. Iron can be directed to the mitochondria for biosynthesis of heme and iron-containing metabolic enzymes or routed for storage bound to ferritin in the liver^22,23^. Conversely, in times of iron limitation such as during lactational fasting, ceruloplasmin and other ferroxidases facilitate iron mobilization from endogenous stores^22^.

This study aimed to determine whether high demand for iron during development in diving mammals drives fitness trade-offs centering around this essential micronutrient, and to determine whether iron transfer during lactation poses longer-term costs to the females. Females that provide more iron to their growing pup would have less iron for their own heme production which may limit dive behaviors and foraging strategies. In this study, we tracked iron stores and hemoprotein levels throughout the lactation and postweaning periods in adult female Weddell seals (*Leptonychotes weddellii*) to determine the magnitude of iron provisioning during lactation and costs to the female. To demonstrate the behavioral consequences of iron transfer during lactation, dive durations were also recorded throughout the year.

Any life history trade-offs centering on iron dynamics may be particularly pronounced in this species because Weddell seals are exceptional divers with high hemoprotein concentrations^24^. Weddell seals routinely make dives 20 min in duration, and the longest recorded dive for the species is 96 min^25^. Weddell seals also have a relatively long lactation period for a phocid seal (6–7 weeks) and although some individuals engage in supplemental foraging towards the end of the lactation period^26,27^, females rely primarily on stored energetic and micronutrient capital to provision their offspring^28^. During late summer, post-partum females spend more time intensively foraging after weaning their pups than females that did not give birth (skip-breeders)^29^, but all females continue making increased foraging efforts for the remainder of the year to recuperate mass lost in support of reproduction and/or the annual molt^30,31^.

Here we report that numerous indices of iron mobilization are all up-regulated during the lactation period in post-partum females. Iron mobilization directly correlates with the amount of iron females are able to incorporate into their milk for transfer to the pup, and deeper-diving marine mammals have much higher milk iron concentrations than terrestrial mammals. Offload of large amounts of iron hinders female Weddell seals’ ability to maintain their own endogenous heme stores, and post-partum females have shorter dive durations following weaning than skip-breeders. Together, this study demonstrates that high iron demand during lactation ultimately leaves dive capacity as a cost of reproduction in a marine mammal.

## Results

### Post-partum females rapidly mobilize iron during lactation

We compared indices of iron mobilization, heme stores, and TBO_2_ at the beginning (7 days post-partum; dpp) and end (35 dpp) of lactation as well as during the post-weaning period (95 dpp) in adult female Weddell seals during the austral summer from 2010–2017. To confirm whether physiologic changes in iron mobilization were in fact due to lactation, we also sampled skip-breeders during this same timeframe, in the pupping season (October–December) and late-summer (January–February) (Table 1).

**Table 1:** Study sample size and Weddell seal body composition.

	Skip-breeders		Post-partum females			Effect of reproductive class
	Early-summer (Lactation Period)	Late-summer	Early-summer beginning lactation (7 dpp)	Early-summer end lactation (35 dpp)	Late-summer post-weaning (95 dpp)	LME F-statistic; *P*-value
*n* for study	63	85	17	64	62	—
Total Body Mass (kg)	389.9 ± 10.0^a^ *(60)*	342.0 ± 7.5^b^ *(84)*	413.7 ± 13.3^a^ *(16)*	282.0. ± 4.6^c^ *(63)*	312.5 ± 5.8^b^ *(59)*	*F*_4,113_ = 46.7, *P* < 0.001*
Lean Mass (kg)	243.0 ± 6.5^a^ *(56)*	232.1 ± 4.9^b^ *(82)*	266.5 ± 8.8^ab^ *(15)*	192.3 ± 3.2^c^ *(62)*	232.8 ± 4.0^ab^ *(59)*	*F*_4,113_ = 39.6, *P* < 0.001*
Lipid (% Total Body Mass)	37.2 ± 0.6^a^ *(56)*	31.9 ± 0.4^b^ *(82)*	36.3 ± 0.8^a^ *(15)*	31.9 ± 0.5^b^ *(62)*	25.3 ± 0.5^c^ *(59)*	*F*_4,164_ = 74.7, *P* < 0.001*

*Asterisk denotes significant difference among reproductive classes.

Study sample size (*n*) and mean ± SE total body mass, lean mass, and lipid (as %total body mass) for skip-breeding and post-partum adult female Weddell seals across the austral summer lactation and post-weaning periods. Statistical significance was detected using Linear Mixed-Effect models and post-hoc comparisons with Bonferroni correction. Sample size for each measure is shown in parentheses and *different letters* (a,b,c) indicate significant differences in physiological parameter between reproductive classes.

**Fig. 1:**
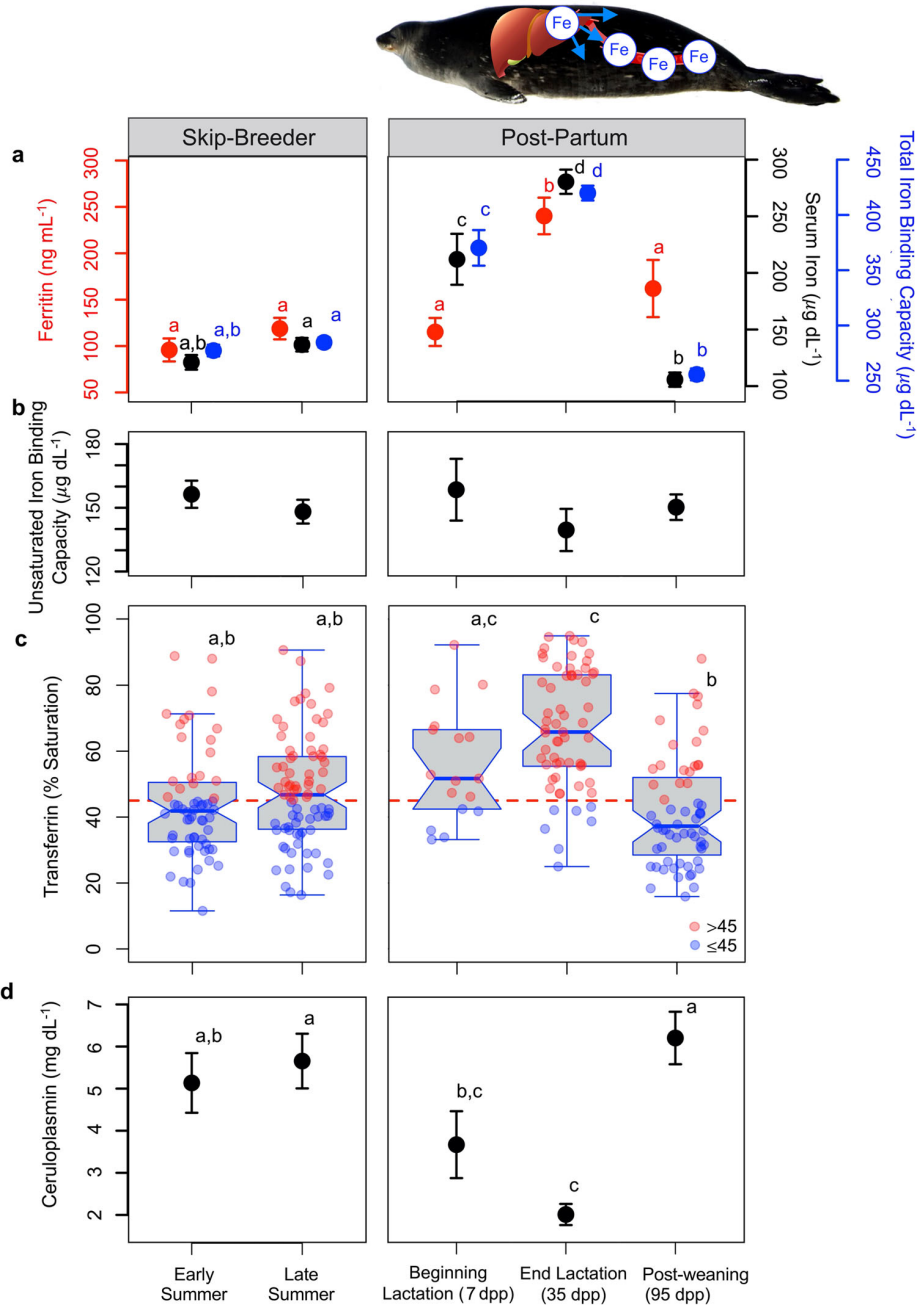
Iron mobilization increased during the austral summer in lactating post-partum females, but not skip-breeders. Mean ± SE (**a**) Plasma ferritin (*red points*), and serum iron (*black points*) and total iron binding capacities (TIBC; *blue points*) increased during lactation, whereas (**b**) unsaturated iron binding capacities (UIBC) did not vary among reproductive classes. **c** This resulted in significantly higher transferrin saturation levels (percentage of sites bound with iron) during lactation in post-partum females as compared with skip-breeders. *Red points* denote transferrin saturation levels that would indicate iron overload in humans (>45%); *blue points* show transferrin saturation levels within the normal range for other mammals; note that points are jittered on the x-axis. In boxplots, boxes encompass the interquartile range, the center line denotes the median, whiskers encompass range of values, and points above or below the whiskers are greater/less than 1.5-times the interquartile range. **d** Plasma ceruloplasmin declined during lactation in female Weddell seals. Statistical significance was detected using Linear Mixed-Effect models and post-hoc comparisons with Bonferroni correction; *Different letters* = significant difference between reproductive classes. Serum iron, TIBC, UIBC, Tf-Sat - Skip-breeder *n*: Early summer = 60, Late summer = 79; Post-Partum *n*: Beginni*n*g Lactation (7 dpp) = 17, End Lactation (35 dpp) = 61, Post-weaning (95 dpp) = 60. Ferritin and Ceruloplasmin - Skip-breeder *n*: Early summer = 16, Late summer = 27; Post-Partum *n*: Beginni*n*g Lactation (7 dpp) = 10, End Lactation (35 dpp) = 16, Post-weaning (95 dpp) = 17. Photo credit: Michelle Shero.

Circulating ferritin concentrations were elevated during lactation in post-partum females, indicating iron mobilization occurred from the liver (Fig. 1; Linear mixed-effect model, LME; reproductive class: *F*_4,44_ = 8.3, *P* < 0.001; See Supplementary Fig. 1 for individual-effects). Additionally, serum iron concentrations were significantly higher by the end of lactation in post-partum females as compared with skip-breeders, also driving significant increases in total iron binding capacity (TIBC, i.e., binding capacity of Tf), and transferrin saturations (Tf-Sat) (Serum iron: *F*_4,64.4_ = 64.6, *P* < 0.001; TIBC: *F*_4,184_ = 134.7, *P* < 0.001; Tf-Sat: *F*_4,178_ = 23.9, *P* < 0.001). That iron stores were mobilized to support lactation was further indicated by the fact that all indices declined in post-partum females after weaning their pups (by 95 dpp; post-hoc test- Ferritin: *z* = –4.0, *P* < 0.001; Serum iron: *z* = –14.6, *P* < 0.001; TIBC: *z* = –20.4, *P* < 0.001; Tf-Sat: *z* = –8.8, *P* < 0.001). In contrast, skip-breeders maintained relatively low iron concentrations throughout the austral summer (all *P* > 0.7). Many female Weddell seals had Tf-Sat levels >45%, which is indicative of iron overload in other mammalian species^32–34^ (31.7% of early-summer skip breeders; 54.4% of late-summer skip breeders; 70.6% of 7 dpp females; 88.5% of 35 dpp females; 31.7% of 95 dpp females). Unsaturated iron binding capacity (UIBC) did not differ among reproductive classes (*F*_4,197_ = 0.8, *P* = 0.532). In post-partum seals, plasma ceruloplasmin concentrations declined significantly during lactation and increased again post-weaning (*F*_4,44_ = 21.8, *P* < 0.001); however, ceruloplasmin concentrations remained high across the austral summer in skip-breeders.

### Serum iron concentrations correlate with milk iron

**Fig. 2:**
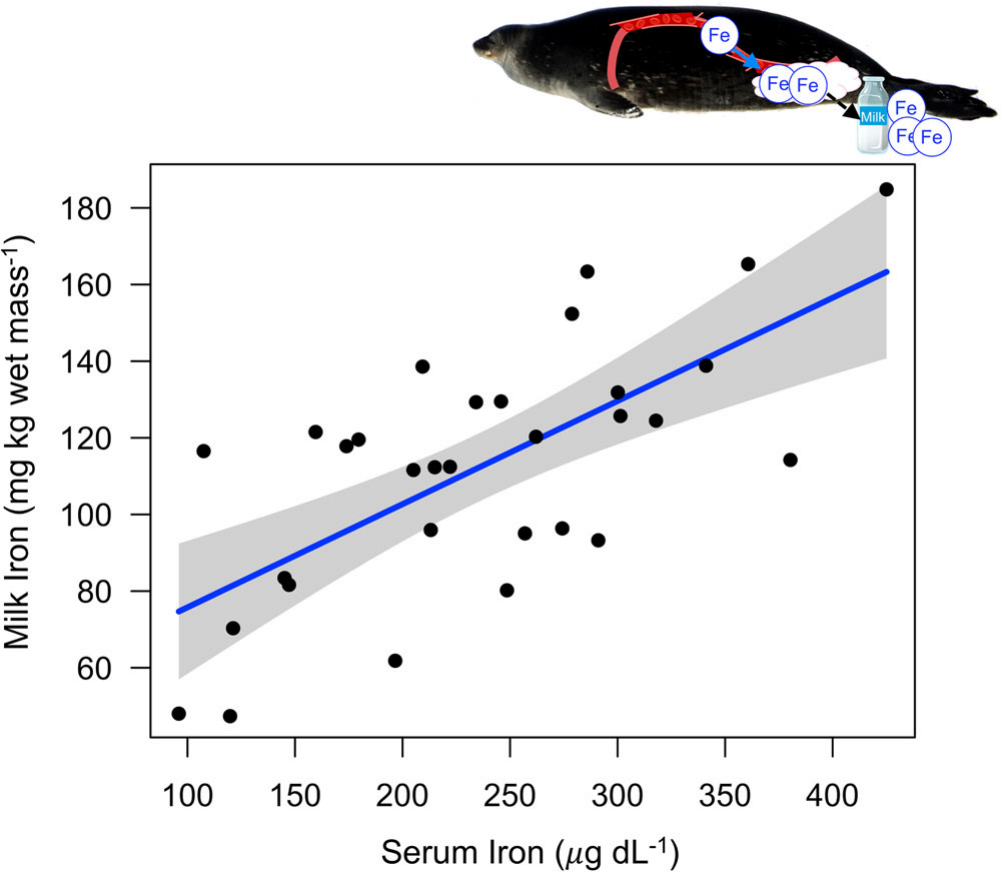
Females with higher serum iron concentrations incorporated more iron into their milk. Linear regression demonstrating that female Weddell seals with greater circulating serum iron levels incorporated more iron into their milk. Gray bands depict the 95% confidence interval around the regression model line (center of error bands). The same relationship was also observed with transferrin-saturation. *n* = 31. Photo credit: Michelle Shero.

Females with higher serum iron concentrations and Tf-Sat were able to incorporate more iron into their milk for transfer to offspring (Fig. 2; Linear regression, Serum Iron: *F*_1,29_ = 25.3, *P* < 0.001, *r*^2^ = 0.465; Tf-Sat: *F*_1,29_ = 20.5, *P* < 0.001, *r*^2^ = 0.414), and Weddell seal milk iron concentrations were exceptionally high relative to other mammals (overall mean ± SE: 112.4 ± 5.9 mg kg wet mass^−1^). There was no difference in milk iron content between the beginning and end of lactation (*F*_1,29_ < 0.001, *P* = 0.988).

### Hemoprotein concentrations decrease only in post-partum seals

Transferring large quantities of iron to the pup through milk hindered post-partum females’ ability to maintain their own endogenous heme stores. Such patterns were not observed in skip-breeders which did not experience the iron demands associated with offspring-rearing.

**Fig. 3:**
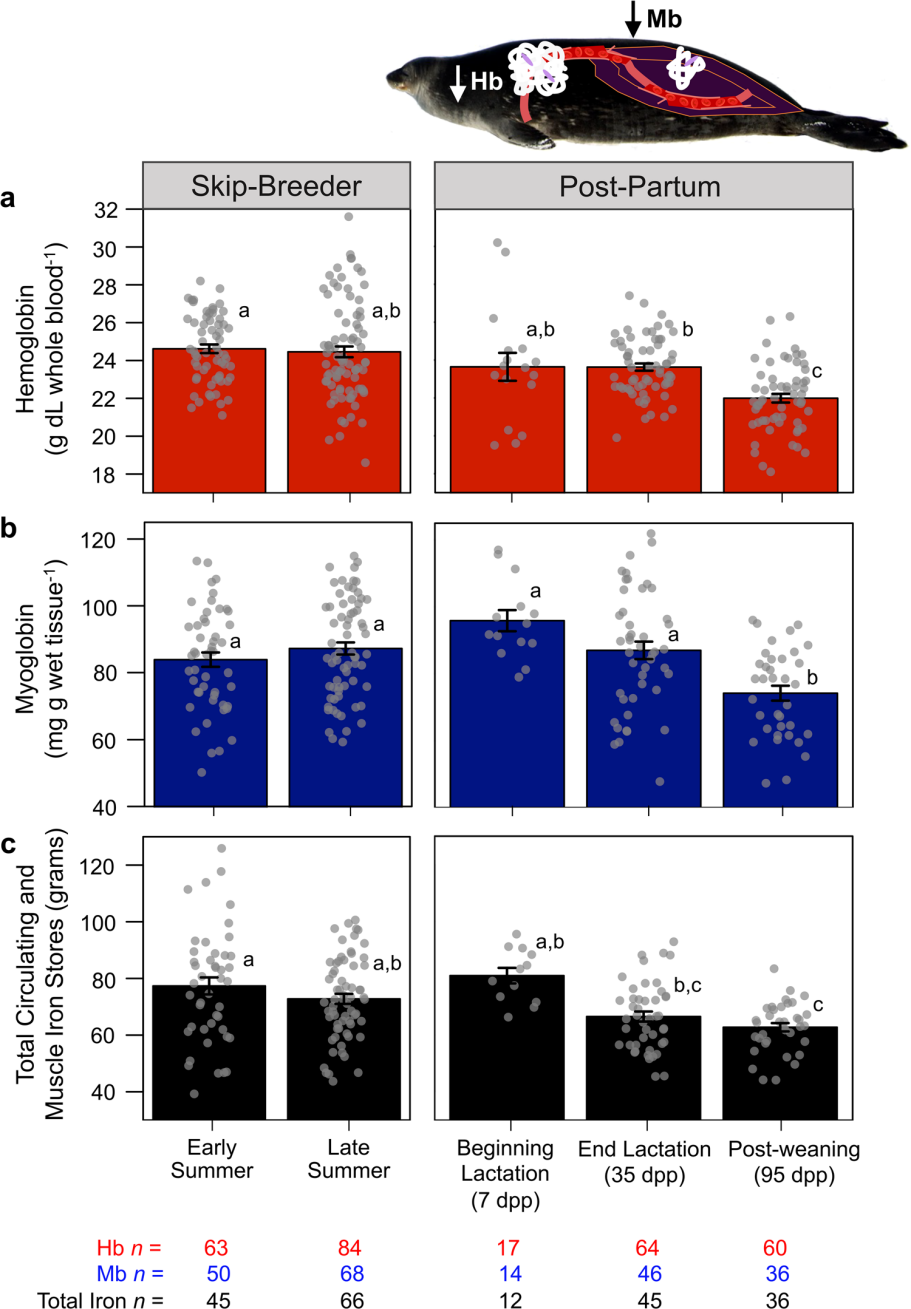
Hemoprotein concentrations and iron stores declined in post-partum females but not skip-breeders. Mean ± SE (**a**) blood hemoglobin concentrations, (**b**) *longissimus dorsi* myoglobin concentrations, and **c** calculated total circulating and muscle iron stores significantly declined in post-partum female Weddell seals but not in skip-breeders. Statistical significance was detected using Linear Mixed-Effect models and post-hoc comparisons with Bonferroni correction; *Different letters* = significant difference between reproductive classes. Photo credit: Michelle Shero.

Skip-breeders had higher blood hematocrit, Hb, and mean corpuscular hemoglobin concentration (MCHC) throughout the study as compared to post-partum females (Table 2). Post-partum females had low hematocrit levels immediately following parturition (7 dpp), and after a transient recovery (at 35 dpp; LME Bonferroni post-hoc: *z* = 3.6, *P* = 0.003), again had lower hematocrit levels after weaning their pups (*z* = –4.8, *P* < 0.001). This was accompanied by a significant decline in blood Hb concentrations by late summer (Fig. 3a; overall LME *F*_4,147_ = 17.1; *P* < 0.001) that was apparent in post-partum females (7 dpp vs. 95 dpp post-weaning: *z* = –3.0, *P* = 0.028; 35 dpp vs. 95 dpp post-weaning: *z* = –5.2, *P* < 0.001) but not in skip-breeders (*z* = 1.0, *P* = 0.867). Similarly, late-summer MCHC was lower for post-partum females than skip-breeders (*z* = 3.1, *P* = 0.017). Post-partum females maintained higher plasma volume (as a proportion of lean body mass) than skip-breeders throughout the study (Table 2). However, post-partum females exhibited a significant decline in blood volume per kg lean mass during late summer after weaning their pups (*z* = –10.1, *P* < 0.001), while skip-breeders maintained a constant lean mass-specific blood volume across the austral summer. Along with experiencing declines in multiple hematological parameters after weaning, post-partum females also displayed a significant decrease in muscle Mb concentrations in the primary locomotor muscle (*Longissimus dorsi*). Post-weaning females (95 dpp) had lower Mb concentrations than all other reproductive groups (Fig. 3b; overall LME: *F*_4,179_ = 6.8, *P* < 0.001). Together, this resulted in a significant decline in total iron stores in circulation and in the muscle in post-partum females, while skip-breeders maintained their iron stores across the austral summer (Fig. 3c; overall LME: *F*_4,89_ = 5.8, *P* < 0.001).

**Table 2:** Shifts in Weddell seal hematological parameters and muscle biochemistry associated with lactation.

	Skip-breeders		Post-partum females			Effect of reproductive class
	Early-summer (lactation period)	Late-summer	Early-summer beginning lactation (7 dpp)	Early-summer end lactation (35 dpp)	Late-summer post-weaning (95 dpp)	LME F-statistic; *P*-value
Hematology						
Hematocrit (%)	62.8 ± 0.6^a^ *(63)*	61.1 ± 0.5^ab^ *(84)*	56.2 ± 1.6^c^ *(17)*	60.4 ± 0.4^b^ *(64)*	57.0 ± 0.6^c^ *(61)*	*F*_4,181_ = 18.7; *P* < 0.001*
MCHC (%)	39.3 ± 0.3^ab^ *(63)*	39.8 ± 0.2^a^ *(83)*	39.9 ± 0.7^ab^ *(15)*	39.1 ± 0.2^ab^ *(64)*	38.8 ± 0.2^b^ *(60)*	*F*_4,151_ = 2.6; *P* = 0.039*
Red Blood Cells (10^6^ *μ*L^−1^)	3.94 ± 0.06^a^ *(48)*	3.78 ± 0.06^a^ *(82)*	3.63 ± 0.17^ab^ *(12)*	3.83 ± 0.05^a^ *(64)*	3.54 ± 0.05^b^ *(55)*	*F*_4,168_ = 7.5; *P* < 0.001*
Plasma Volume (L)	22.8 ± 0.8^a^ *(60)*	22.7 ± 0.6^a^ *(82)*	27.7 ± 1.0^b^ *(16)*	21.7 ± 0.5^a^ *(63)*	24.9 ± 0.6^b^ *(56)*	*F*_4,123_ = 17.4, *P* < 0.001*
Plasma Volume (%LBM)	9.33 ± 0.16^a^ *(56)*	9.70 ± 0.14^b^ *(80)*	10.44 ± 0.19^bcd^ *(15)*	11.32 ± 0.19^c^ *(62)*	10.54 ± 0.15^d^ *(55)*	*F*_4,144_ = 24.1, *P* < 0.001*
Blood Volume (L)	61.9 ± 2.1^a^ *(60)*	58.1 ± 1.5^b^ *(82)*	62.9 ± 2.2^ab^ *(16)*	55.2 ± 1.3^ab^ *(63)*	57.1 ± 1.4^ab^ *(56)*	*F*_4,128_ = 3.6, *P* = 0.009*
Blood Volume (%LBM)	25.5 ± 0.5^a^ *(56)*	25.1 ± 0.4^a^ *(80)*	23.5 ± 0.6^a^ *(15)*	28.7 ± 0.5^b^ *(62)*	24.1 ± 0.4^a^ *(55)*	*F*_4,150_ = 29.8, *P* < 0.001*
Muscle Biochemistry						
CS (IU g wet tissue^−1^)	16.0 ± 0.6^ab^ *(47)*	15.3 ± 0.4^ab^ *(64)*	13.3 ± 1.3^bc^ *(14)*	12.9 ± 0.5^c^ *(41)*	17.4 ± 0.7^a^ *(27)*	*F*_4,152_ = 8.3, *P* < 0.001*
HOAD (IU g wet tissue^−1^)	29.2 ± 1.4^ab^ *(48)*	26.1 ± 1.1^ab^ *(65)*	22.9 ± 2.4^ab^ *(14)*	24.7 ± 1.5^a^ *(43)*	30.7 ± 2.0^b^ *(29)*	*F*_4,135_ = 3.1, *P* = 0.018*
LDH (IU g wet tissue^−1^)	693.0 ± 19.6^a^ *(48)*	506.9 ± 13.6^bc^ *(65)*	592.8 ± 29.0^b^ *(14)*	509.3 ± 20.7^bc^ *(43)*	459.7 ± 17.7^c^ *(29)*	*F*_4,135_ = 24.9, *P* < 0.001*
LDH:CS	46.1 ± 2.1^a^ *(47)*	35.3 ± 1.7^b^ *(64)*	49.9 ± 5.2^a^ *(14)*	42.4 ± 2.7^ab^ *(41)*	27.3 ± 1.6^c^ *(27)*	*F*_4,154_ = 13.9, *P* < 0.001*
CS:HOAD	0.58 ± 0.02 *(47)*	0.63 ± 0.02 *(64)*	0.60 ± 0.04 *(14)*	0.60 ± 0.04 *(41)*	0.61 ± 0.03 *(27)*	*F*_4,164_ = 0.9, *P* = 0.457

*Asterisk denotes significant difference among reproductive classes.

Mean ± SE Weddell seal hematology (hematocrit, mean corpuscular hemoglobin concentration (MCHC), red blood cell counts), plasma and blood volume (as Liters or scaled to lean body mass, LBM), and *longissimus dorsi* skeletal muscle aerobic (Citrate synthase, CS; β-Hydroxyacyl CoA dehydrogenase, HOAD) and anaerobic (Lactate dehydrogenase, LDH) enzyme activities in skip-breeding and post-partum females across the austral summer lactation and post-weaning periods. Statistical significance was detected using Linear Mixed-Effect models and post-hoc comparisons with Bonferroni correction. Sample size for each measure is shown in parentheses and *different letters* (a,b,c) indicate significant differences in physiological parameter between reproductive classes.

Decreased Mb-O_2_ carrying capacities were also reflected in significantly lower aerobic citrate synthase activity (CS; marks entrance to the TCA cycle and is proportional to metabolic rate^35^) at the end of lactation when compared to skip-breeders (Table 2; post-hoc test: all *P* < 0.02); however, muscle aerobic CS activity increased over the late-summer postweaning period (35 dpp vs 95 dpp: *z* = 5.2, *P* < 0.001) such that it was again equivalent to skip-breeders (all *P* > 0.1). Post-partum female muscles also showed an increase in lipid reliance (increased *β*-hydroxyacyl-CoA-dehydrogenase; HOAD) post-weaning (35 dpp vs 95 dpp: *z* = 2.9, *P* = 0.036), but there was no effect of reproductive class on CS:HOAD ratios. Both skip-breeders and post-partum females also exhibited significant decreases in muscle anaerobic capacities as evident by lactate dehydrogenase (LDH) activities and LDH:CS ratios, and post-partum females ended the late-summer postweaning period with the lowest anaerobic capacities.

### Hemoprotein loss in post-weaning females

**Fig. 4:**
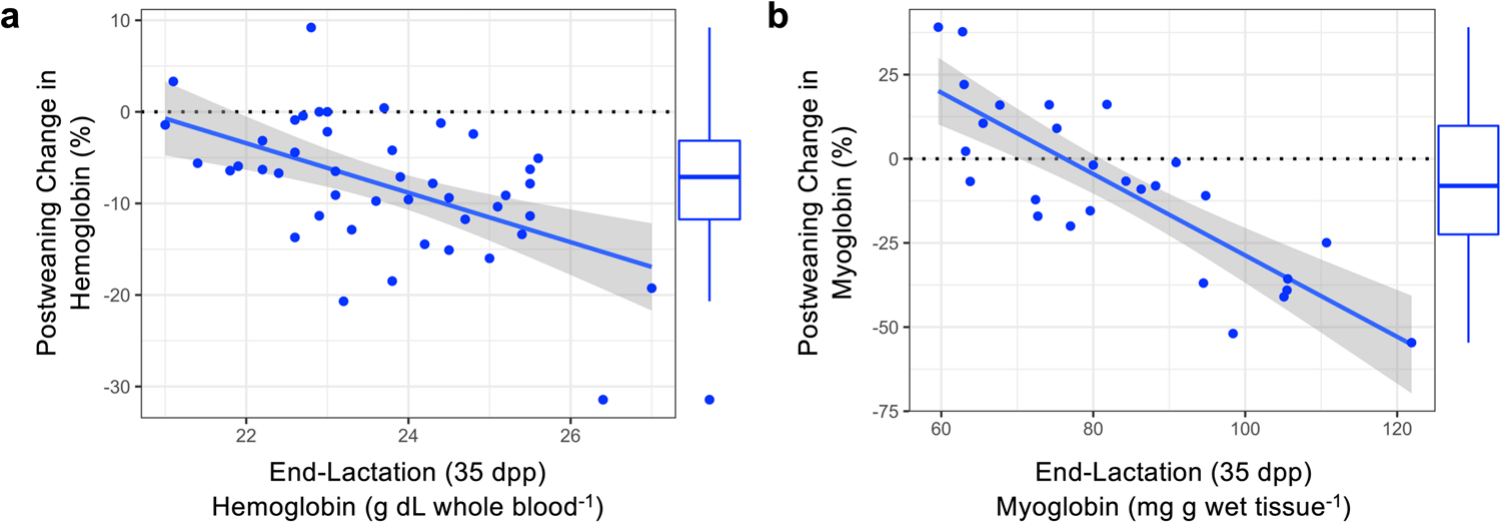
Hemoprotein concentration at the end of lactation determined the magnitude of hemoprotein loss post-weaning. Female Weddell seals with higher (**a**) blood hemoglobin and (**b**) muscle myoglobin at the end of lactation (35 dpp) exhibited greater declines in hemoprotein concentrations post-weaning (by 95 dpp) on a percent basis (See Supplemental material for changes in absolute values). Gray bands depict the 95% confidence interval around the regression model line (center of error bands); boxplots depict the distribution of changes in hemoprotein concentrations where boxes encompass the interquartile range, the center line denotes the median, whiskers encompass range of values, and points above or below the whiskers are greater/less than 1.5-times the interquartile range. *n* for Hb = 45; Mb = 27.

Female heme stores continued to decline after weaning their pups. Females that had greater blood Hb concentrations during late lactation (35 dpp) could afford to provide more iron to offspring and exhibited a greater decline in Hb post-weaning on an absolute (Linear Regression: Supplementary Fig. 2; *F*_1,43_ = 21.1, *P* < 0.001, *r*^2^ = 0.329) and percent-basis (Fig. 4a; *F*_1,43_ = 16.8, *P* < 0.001, *r*^2^ = 0.281). Females that had higher Hb concentrations during late lactation also had greater muscle Mb (*F*_1,44_ = 5.0, *P* = 0.031), and proceeded to lose more Mb post-weaning (absolute: *F*_1,25_ = 72.8, *P* < 0.001, *r*^2^ = 0.745; Fig. 4b; percent: *F*_1,25_ = 52.4, *P* < 0.001, *r*^2^ = 0.677). As a result, by the end of the summer all post-weaning females had equivalent heme stores regardless of the Hb or Mb levels that females had at the end of lactation (i.e., there was no relationship between Hb (or Mb) at 35 dpp and 95 dpp; Linear regression, Hb: *F*_1,43_ = 2.7, *P* = 0.105; Mb: *F*_1,25_ = 1.6, *P* = 0.212).

### Total body oxygen stores and cADLs only decline in post-partum seals

**Fig. 5:**
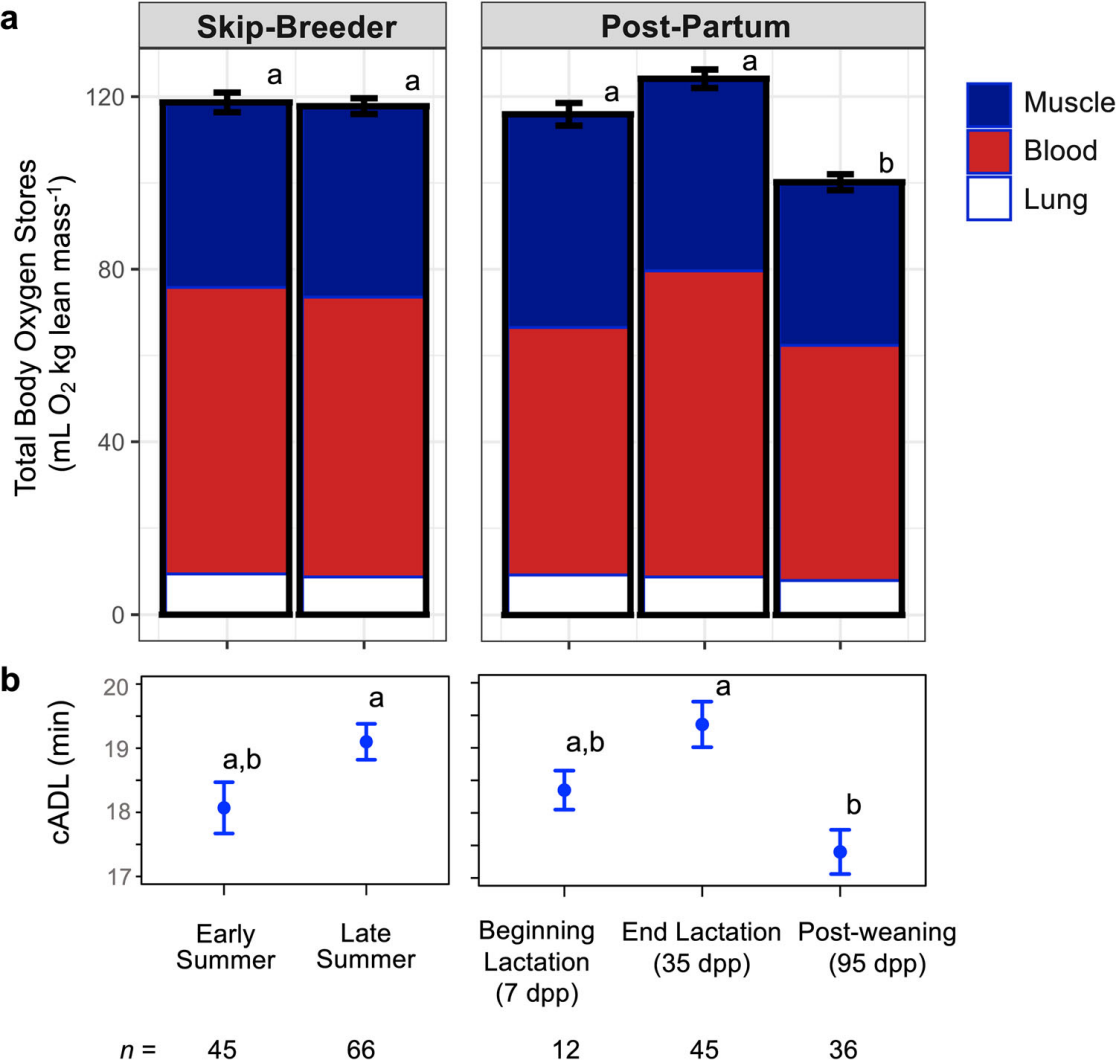
Decreased TBO_2_ and cADLs after lactation. Mean ± SE (**a**) Weddell seal total body oxygen stores scaled to lean body mass (and divided into muscle, blood, and lung compartments) and **b** the calculated aerobic dive limit declined significantly by the end of the austral summer only in post-partum females. Statistical significance was detected using Linear Mixed-Effect models and post-hoc comparisons with Bonferroni correction; *Different letters* = significant difference in TBO_2_ and the cADL between reproductive classes.

Taken together, the significant declines in blood volume and in blood and muscle heme proteins resulted in significantly lower TBO_2_ stores during the late-summer period after post-partum females weaned their pups (Fig. 5; See Supplementary Table 1 for absolute O_2_ stores and DMR). When scaled to lean mass, post-partum female blood O_2_ stores were significantly lower right after parturition (7 dpp) and after weaning their pups (95 dpp) when compared to all other reproductive classes (overall LME: *F*_4,151_ = 30.3, *P* < 0.001). Lean mass-specific muscle O_2_ and TBO_2_ stores were also significantly lower in post-partum females after weaning their pups (95 dpp) than in all other reproductive classes (Muscle O_2_: *F*_4,181_ = 6.9, *P* < 0.001, post-hoc tests all *P* < 0.010; TBO_2_: *F*_4,172_ = 18.0, *P* < 0.001, post-hoc tests all *P* < 0.010). The aerobic dive limit was calculated (cADL) as TBO_2_ divided by DMR. Post-partum female cADLs were significantly lower after females weaned their pups when compared to the end of lactation and late-summer skip-breeders (decreased by 10.3%, LME: *F*_4,138_ = 5.6, *P* < 0.001, post-hoc tests all *P* < 0.005).

### Dive durations decline in post-partum females but not skip-breeders

**Fig. 6:**
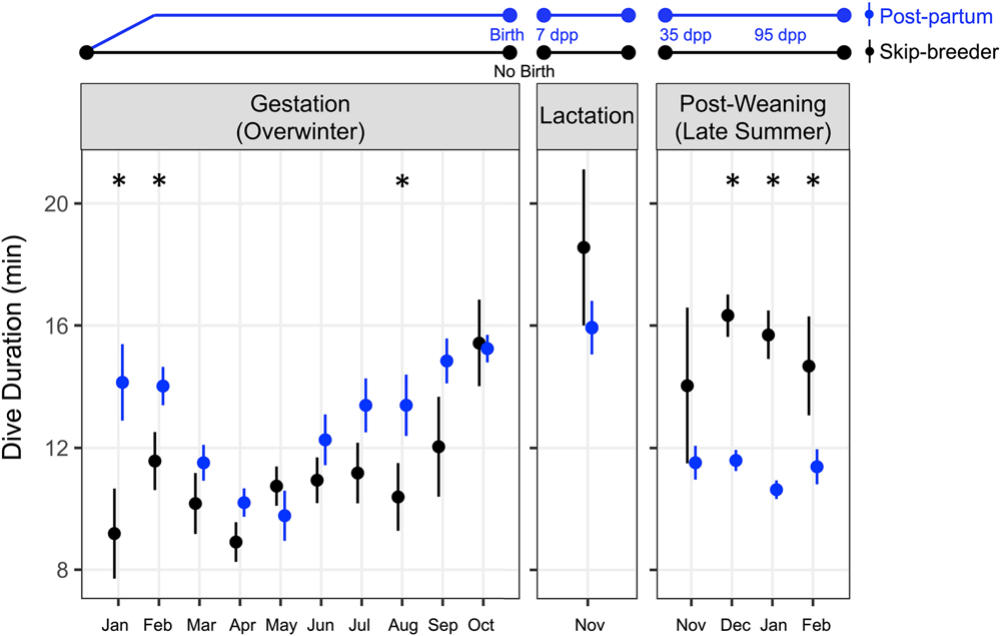
Year-round changes in skip-breeder and post-partum Weddell seal dive durations. Weighted mean ± SE dive duration of female Weddell seals that were (*left*) instrumented as skip-breeders in years 2010–2012. Some females returned after the winter foraging period and gave birth (blue, *n* = 12) while others failed to produce a pup (black, *n* = 8); data redrawn from Shero et al. 2018^31^, and are the same seals included in this study. (*Middle*) Dive duration across lactation; skip-breeder value from Shero et al. 2018, lactating female value from Sato et al. 2003^101^ (*n* = 8). During late-summer (*right*), post-partum females were instrumented at 35 dpp in years 2013–2017 and dive durations decreased during the post-weaning period relative to durations during the lactation period (*n* = 40), whereas skip-breeder dive durations did not (*n* = 19); data from Beltran et al. 2021^30^ and are the same seals included in this study. Only animals with >50 dives per month were included in the weighted mean. Generalized Additive Mixed-effect Models were used to test for significance; *Asterisk* = significant difference between reproductive classes.

Reduced hemoprotein concentrations, TBO_2_, and the cADL occurred concomitant with shifts in late-summer dive behavior in post-partum females. Indeed, post-partum female dive durations declined, and late-summer dives were the shortest in duration when compared to the rest of the year (Fig. 6). In contrast, skip-breeding females (that did not have the high iron demands of supporting a pup and did not exhibit declines in TBO_2_ stores and the cADL) had significantly longer dive durations than post-partum seals (GAMM Post-weaning December: *F* = 41.6, *P* < 0.001; January: *F* = 50.2, *P* < 0.001; February: *F* = 5.7, *P* = 0.017), and skip-breeders made their longest dives of the year during the summer foraging period.

## Discussion

Reduced hemoprotein concentrations and TBO_2_ stores following lactation in adult female Weddell seals reveals a cost of reproduction in a diving mammal that has been previously unexplored. While it is well-known that marine mammals exhibit a dramatic loss of body mass and lipid reserves during lactation^4–6,28^, this is the first study to demonstrate large iron offload via the milk such that females were unable to maintain their own endogenous heme stores. The decline in aerobic dive capacities following lactation observed in this study indicates there is a fitness tradeoff associated with the allocation of finite micronutrient resources. Pups would likely benefit from greater iron acquisition to meet the demands of erythron expansion, production of hemoproteins, and TBO_2_ maturation^12,36,37^ as this would enhance breath-hold abilities at the initiation of independent foraging when mortality rates are generally high in pinniped pups (~50% in the first year across species)^38–40^. If sufficient iron provisioning increased offspring survivorship it would also increase the female’s fitness. However, there may be carryover effects of exceptional iron transfer from one reproductive cycle to the next. Because breath hold capacities are so tightly linked with O_2_-carrying heme proteins in marine mammals, post-weaning declines in female foraging ability following lactational iron transfer could potentially hinder energy allocation towards future reproduction^31,41–43^.

Elevated circulating iron, TIBC, Tf-Sat, and ferritin concentrations during lactation all indicate rapid mobilization of iron from endogenous stores^21,44^. Pinnipeds have exceptionally high Tf-Sat during lactation^11,45^ (up to 95% in Weddell seals in *this study*; mean 7 dpp: 55.9 ± 4.2%, 95 dpp: 66.8 ± 2.3%) compared to terrestrial mammals and humans during lactation (humans, second week: 15.7 ± 9.0%; fourth month: 24.4 ± 8.9%^46^) at levels that would typically lead to reactive oxygen species (ROS) generation, cellular damage, and organ dysfunction. Weddell seals had 3–4× higher ferritin concentrations than other lactating pinnipeds (hooded, *Cystophora cristata*; harp, *Pagophilus groenlandicus*; and harbor seals, *Phoca vitulina*)^11^ suggesting large liver iron stores to draw from^47^. While ceruloplasmin generally increases during iron mobilization and its primary role as a ferroxidase prevents oxidative stress^44^, high ceruloplasmin also facilitates the reincorporation of iron into ferritin for storage in the liver^22,48^. Thus, instead of shuttling labile iron to the liver^49^, cellular mechanisms such as decreased ceruloplasmin during lactation may serve to prioritize mobilization of iron into the milk in diving mammals. In other mammals, small quantities of iron are lost by the exfoliation of gastrointestinal cells, bile, and urine, but there is no mechanism for substantial iron excretion^21^. Rather, iron homeostasis is achieved through regulating the distribution and recycling of iron among tissues^21^. Diving mammals may have a unique ability to transfer immense quantities of iron to the mammary tissue and milk for offload.

**Fig. 7:**
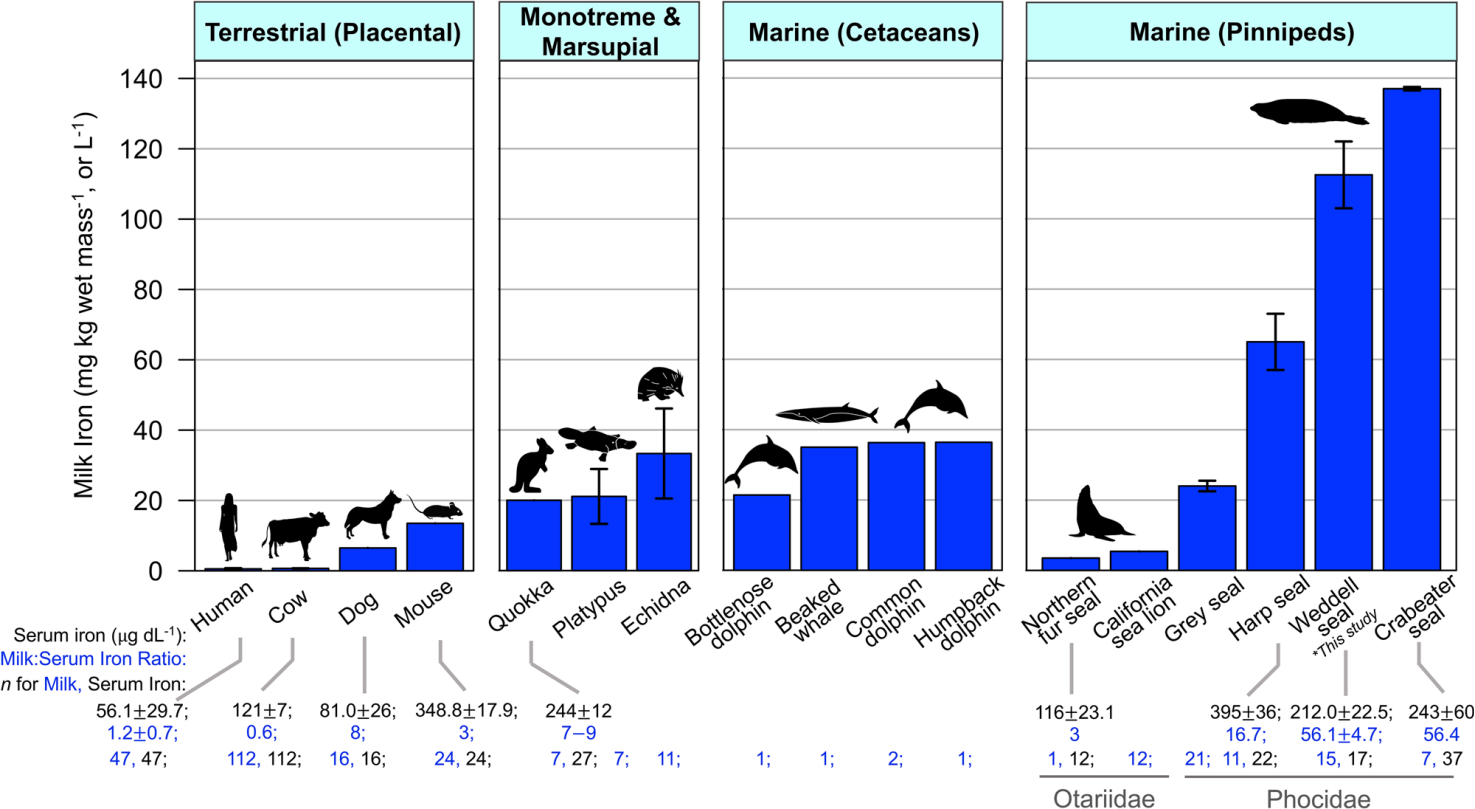
Milk iron concentrations (mean ± SE) across a range of terrestrial and marine mammals. (*Below; black text*) Mean ± SE serum iron concentrations are shown, and (*blue text*) the milk:serum iron ratio indicates the magnitude that each species prioritizes iron mobilization into the milk to transfer to offspring. Data are from sources: Human (*Homo sapiens*, milk iron at 2^nd^ week lactation: 0.55 ± 0.20 mg L^−1^; 4^th^ month lactation: 0.38 ± 0.18)^46^, Cow (*Bos taurus*)^51^, Dog (*Canis lupus*)^104^, Mouse (*Mus musculus*)^50,56^, Quokka (*Setonix brachyurus*, milk iron at 40–59 dpp: 12 mg L^−1^; 60–150 dpp: 20)^52^, Platypus (*Ornithorhynchus anatinus*)^105^, Echidna (*Tachyglossus aculeatus*)^105^, Bottlenose dolphin (*Tursiops truncatus*)^106^, Stejneger’s beaked whale (*Mesoplodon stejnegeri*)^107^, Common dolphin (*Delphinus delphis*)^106^, Humpback dolphin (*Sousa plumbea*)^106^, Northern fur seal (*Callorhinus ursinus*)^45,108^, California sea lion (*Zalophus californianus*)^109^, Grey seal (*Halichoerus grypus*, milk iron at 0–2 dpp: 24 ± 1.5 mg L^−1^; 15–17 dpp: 17 ± 1.1)^110^, Harp seal (*Pagophilus groenlandicus*)^11^, Weddell seal (*Leptonychotes weddellii*; *this study, milk iron at 7 dpp: 112.5 ± 9.5 mg L^−1^; 35 dpp: 112.3 ± 7.4), Crabeater seal (*Lobodon carcinophagus*)^111,112^. For species with documented shifts in milk iron across lactation, the maximum is shown in the barplot. Milk:Serum iron ratios were calculated using reported values from separate studies or reported ranges, except for the human and Weddell seal for which the ratios could be calculated in the same individuals. More terrestrial eutherian species can be found in Casey et al.^113^. The subset of species included here encompass the full range of milk iron concentrations in terrestrial eutherian species. Note that serum iron values are only reported if available for a species specifically during lactation.

Milk iron concentrations in terrestrial placental mammals are typically ~0.3–15 mg kg^−1^_,_ which is ~0.5–3× circulating levels (Fig. 7)^46,50,51^. Monotremes and marsupials have higher milk iron concentrations (12–33 mg kg^−1^) which has been postulated to be due to low endogenous iron stores at birth in these species after an exceptionally short gestation^52^. Weddell seal milk iron concentrations in this study were ~10–100× higher than terrestrial eutherian mammals, ~3–9× higher than monotremes and marsupials, and also substantially higher than all values reported in other marine mammal species^11^ except the closely-related crabeater seal (*Lobodon carcinophagus*). Weddell seal milk iron concentrations were also ~50× higher than that of the serum, further supporting that this species has adaptations for highly effective transport of iron into the mammary gland.

Milk intake for Weddell seal pups^53^ ranges from 2.75–5.46 L milk day^−1^ and based on mean milk iron concentrations determined in this study, females would be transferring 309–614 mg Fe day^−1^ to the pup. This is an exceptional rate of transfer relative to terrestrial mammals, and is ~15–100× higher than the recommended iron intake (6 mg Fe day^−1^ for infants; 18 mg Fe day^−1^ for women; 8 mg Fe day^−1^ for men), and 8–15× higher than doses that exceed daily upper limits and result in iron toxicity for humans (40 mg Fe day^−1^)^54^ despite relatively similar body size (adult human: ~70 kg; nursing Weddell seal pup: ~25–100 kg)^28^. The mechanisms allowing pinnipeds to mobilize such large quantities of iron without pathology of iron overload remain unknown. While bioavailability of milk iron has yet to be determined in pinnipeds, it is relatively low in terrestrial mammals (~10% for bovine calves, ~49% in human infants^55^) and thus it is unlikely that the pup could ultimately utilize all the milk iron provided. Further study is needed to determine whether selective pressures to develop large endogenous heme stores have resulted in more effective iron uptake through the gastroenteral cells or greater tissue incorporation rates to enhance female-to-pup iron transfer efficiencies in diving mammals.

**Fig. 8:**
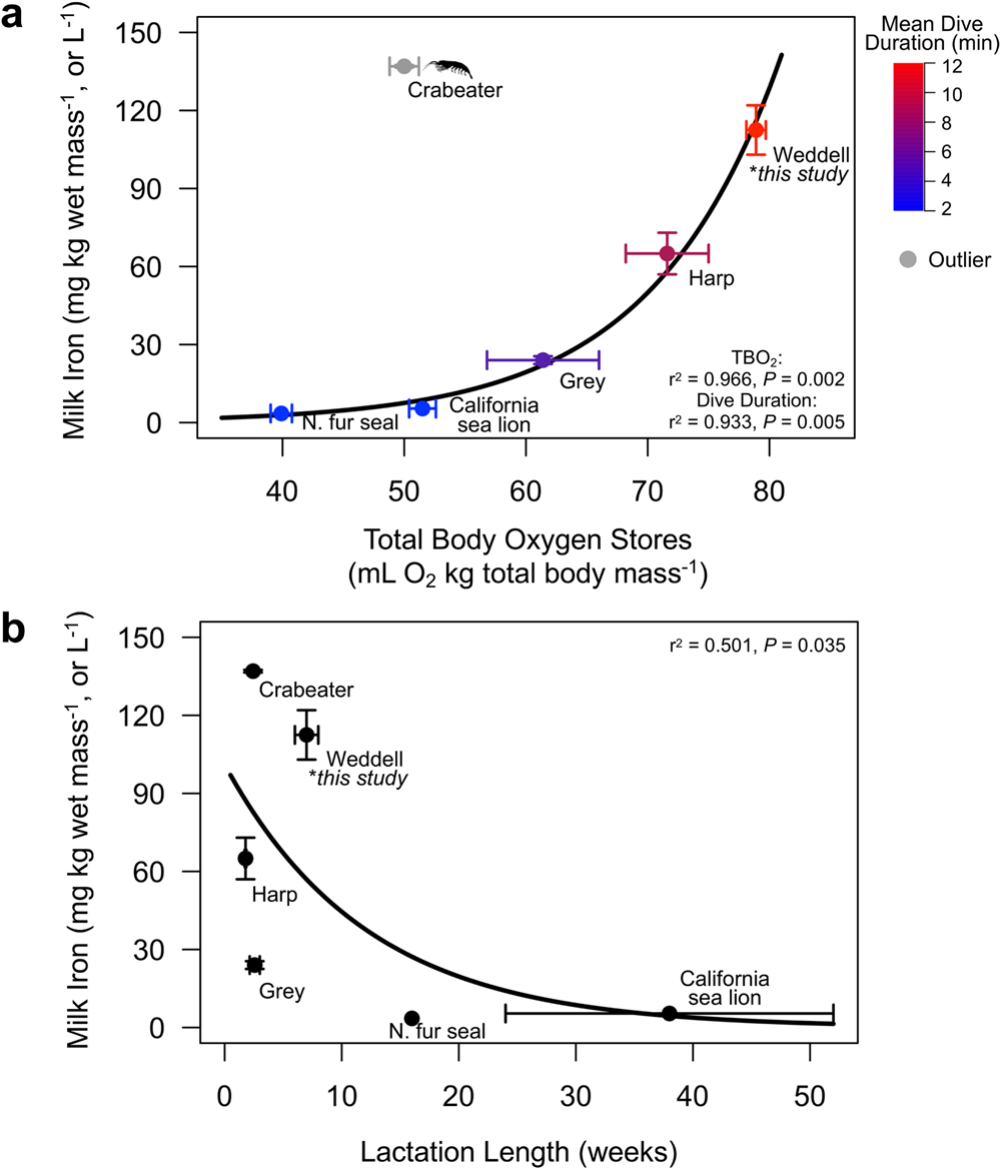
Potential selective pressures for high milk iron in pinnipeds. **a** Mean ± SE milk iron concentrations were significantly higher in species with larger total body oxygen (TBO_2_) stores^12,16,61,62,64^ that made longer dives^31,57–60,63^. Note that the crabeater seal was an outlier that was excluded from the model, and is the only species that primarily consumes krill. Total body oxygen stores were scaled to total body mass, as body composition data were not available for all studies to have scaled O_2_ to lean mass across species. Milk iron values from the beginning of lactation were used for species with multiple sampling timepoints. **b** Pinniped species with shorter lactation periods^4,12,28,61,62,112^ also had higher milk iron concentrations. Error bars show the accepted range in lactation length with points showing the center value. General Linear Models (Gamma family, log link) were used to test for statistical significance between life history traits and milk iron concentration. Sample sizes for milk iron concentration, TBO_2_, and dive duration for the northern fur seal: 1, 6, 7; California sea lion: 12, 37, 7; grey seal: 21, 10, 14; harp seal: 11, 6, 16; Weddell seal: 15, 57, 20; crabeater seal: 7, 35, 34.

The amount of iron that is incorporated into the female’s milk may depend on multiple factors such as the magnitude of endogenous stores^50,56^, the duration of the lactation period, and/or lactation strategy (i.e., capital-, income-, or mixed capital-income breeding). For example, Weddell and harp seals routinely make longer and deeper dives (Fig. 8a; Weddell seal: 11.3 mins duration, 154 m depth; harp seal: 8.1 mins, 141 m)^31,57^ than grey seals (*Halichoerus grypus*, 5.5 mins duration, 49 m depth)^58^ and the otariids (2.2 and 3.1 mins duration; 68 and 93 m depth for northern fur seals, *Callorhinus ursinus* and California sea lions, *Zalophus californianus*, respectively)^59,60^, and correspondingly have greater hemoprotein concentrations (Weddell seal: [Hb] = 23.7 g dL^−1^, [Mb] = 84.8 mg g wet tissue^−1^, *this study*; harp seal: [Hb] = 22.6, [Mb] = 83.3; grey seal: [Hb] = 19.6, [Mb] = 40; northern fur seal: [Hb] = 12.6, [Mb] = 35.8; California sea lion: [Hb] 16–19, [Mb] = 35–55)^12,16,61,62^ and greater milk iron concentrations. Compared to shorter and shallower divers, Weddell seal pups would require more iron during ontogeny to attain adult dive capacities. However, crabeater seals make relatively short and shallow dives (4.6 mins, 76 m)^63^ and have Hb concentrations similar to other deep-diving phocid species ([Hb] = 23.2 g dL^−1^) but low Mb (37.6 mg g wet tissue^−1^)^64^. Notably, this is the only species that primarily consumes iron-rich krill (*Euphausia superba*)^65^ and likely has much higher dietary iron intake than other pinnipeds which may explain why crabeater seals have even higher milk iron concentrations than Weddell seals in this study.

Lactation strategy may also dictate how iron is provisioned. Marine mammal species with shorter lactation durations typically have milk with higher caloric density to provide sufficient energy to the pup within a short timeframe, and there may also be selective pressures to incorporate more iron into the milk to ensure sufficient transfer to the pup (Fig. 8b). Indeed, species such as the harp and crabeater seals with short nursing periods (harp: 7–12 days; crabeater: ~17 days)^12,66^ have higher milk iron concentrations, particularly when compared with otariids that can spread iron transfer over much longer lactation periods (i.e., northern fur seal: lactation length of ~4 months; California sea lion, ~6–12 months)^4,62^ and had milk iron concentrations comparable to terrestrial eutherian mammals. While the Weddell seal has a longer lactation period (6–7 weeks) than many other phocids, supplemental foraging during lactation (up to 25% of day diving)^67^ and thus additional iron intake, may allow females to transfer more iron to the pup than would otherwise be possible if females were strictly-fasting capital breeders. It is noteworthy that the Antarctic seals had the highest milk iron concentrations among marine mammals. It may be particularly advantageous for these species to equip pups with more iron reserves to utilize at the start of independent foraging, as the Southern Ocean is an iron-limited environment where fish prey resources also have lower iron content than fish in other regions^68,69^. Further comparative studies are needed to discern the selective pressures driving inter-specific variation in lactational iron transfer.

That hemoprotein concentrations were still relatively high at the end of lactation suggests there is a notable lag between iron mobilization and the decline in Hb and Mb concentrations. This may indicate that during the early stages of lactation, endogenous iron stores were sufficient for the females to transfer iron to the pup, and it may not have been until later in lactation after tissue stores were depleted that there were deleterious effects to the dam’s hemoprotein levels. The lag in hemoprotein declines is likely to reflect the time required for production of new erythrocytes (Fe incorporation into erythrocytes takes ~7–10 days; erythrocyte lifespan is ~3–4 months in humans^70^) and protein turnover to occur (half-life of Hb is 50–60 days; Mb is 80–90 days in humans^71^). Erythrocyte and hemoprotein turnover rates have yet to be measured in marine mammals. However, pinnipeds have exceptionally high carboxyhemoglobin and heme oxygenase relative to terrestrial mammals^72,73^, which indicate a greater magnitude of hemolysis and rapid erythrocyte turnover^74,75^. Turnover rates would also influence the time it takes for hemoprotein levels to recover post-weaning.

This study shows that not only are adult female pinnipeds in poor condition post-weaning^28^, but this is concurrent with having compromised dive capacities. Reduced diving activity during lactation followed by short and shallow dives post-weaning would also decrease hypoxia exposure, which stimulates numerous pathways (i.e., NFAT/MEF-2, hypoxia inducible factor (HIF)−1, Sp1) that increase transcription of erythropoietin (EPO), hemoproteins, and glycolytic enzymes^76–80^. Compromised breath-holding abilities are also likely to have downstream effects on iron uptake. With limited hypoxic exposure, lower HIF-1 levels release its inhibitory effects on hepcidin hormone production in the liver. Hepcidin acts to degrade FPN1 and thereby prevents iron from entering circulation^81^. Therefore, downregulation of hypoxic signaling could also contribute to lower Hb, Mb, and TBO_2_ post-weaning through reducing both iron absorption and hemoprotein production. However, skip-breeders also decrease activity levels during the mid-summer period as they undergo their annual molt, which is shifted earlier in the summer relative to post-partum females^29^. Yet skip-breeders do not exhibit the decline in physiologic dive capacities that was observed in post-partum females. Post-weaning females had higher muscle aerobic CS activities (proportional to metabolic flux through the TCA cycle^23,82^) when TBO_2_ stores were lowest, which could potentially serve to offset the reduction in hemoprotein concentrations. Lower LDH activities post-weaning suggests that seals were unlikely to be able to extend dive durations using anaerobic metabolism. Shortly after the annual molt, most Weddell seals leave the vicinity and begin their long overwinter foraging migration at-sea^83^, and thus we were unable to determine precisely when hemoprotein levels recover to pre-parturition levels.

Reduced physiologic dive capacity would typically be detrimental to the recuperation of body mass and lipid stores post-weaning. However, in Weddell seals the impacts of reduced aerobic dive capacities may be mitigated if tightly linked with the seasonal pulse of productivity in the high-latitude Antarctic environment^84–86^. Indeed, post-partum females that exhibit significant declines in heme stores and TBO_2_ late-summer make significantly shorter dives as compared with skip-breeding females that did not undergo the costs of lactation and have maintained their heme and aerobic capacities. The reduction in heme and TBO_2_ occurs simultaneously with the summer phytoplankton bloom in McMurdo Sound^86,87^. The high summer productivity may cause prey to concentrate at shallower depths within the water column^84^, and indeed, Weddell seals overall dive to significantly shallower depths at this time of the year^30^. The fact that post-partum females gained mass during the late-summer period despite their shorter dive durations indicates that they were not too adversely impacted by reduced post-weaning dive capacity. However, the precise temporal matching of decreased dive potential with the seasonal pulse of productivity may make this species more vulnerable to climate regime shifts that would decouple these events.

In summary, we demonstrate that in Weddell seals, exceptional iron mobilization during lactation hinders the female’s ability to maintain steady-state hemoprotein production. Reduced blood Hb and muscle Mb post-weaning resulted in a significant decline in the female’s TBO_2_ stores and this decreased aerobic dive capacity was associated with behavioral shifts. It appears that most marine mammals incorporate more iron into their milk, often by many orders of magnitude, than terrestrial mammals. Females may be essentially ‘transferring their dive capacities’ to offspring. Thus, further investigation is needed to better understand how the environment and preyscape may dictate iron transfer rates, implications that maternal iron transfer has on pup dive ontogeny, and the extent to which lactation hinders heme production and dive capacities in other marine mammal species. While most studies of marine mammal reproduction focus on energetic transfer, we identify a previously unexplored cost of reproduction in a marine mammal: dive capacity.

## Methods

All work in this study complies with ethical regulations, and animal handling protocols were approved by the University of Alaska Anchorage and Fairbanks, and University of California Santa Cruz’s IACUC committees. Research and sample import to the United States were authorized under NMFS MMPA permits: 87-1851-04 and 17411-03. Research activities were also approved through Antarctic Conservation Act permits while at McMurdo Station.

### Animal handling

Adult female Weddell seals were captured in Erebus Bay (~77°S, 165°E) and the Victorialand coastline (76°S, 162°E; south of the Drygalski Ice Tongue) from 2010–2017. To measure shifts in iron stores and heme flux across lactation, females were handled at ~7 days post-partum (dpp; mean: 7.3 ± 1.5; range: 3–16 dpp; at the beginning of lactation in years 2010–2012 only^17^), ~35 dpp (mean: 34.8 ± 0.3; range: 29–44 dpp; end lactation; in years 2013–2017), and ~95 dpp (mean: 96.9 ± 1.4; range: 71–123 dpp; postweaning, during the annual molt, in years 2013–2017). Females were not captured until a few days after parturition to ensure sufficient bonding with the pup prior to handling. Similarly, a standardized timepoint towards the end of lactation was chosen because some females can forage intensively late in lactation, making them difficult to capture just prior to weaning. Animals were all either previously flipper-tagged with a unique identification number, or tagged at handling, to determine birth dates and facilitate recapture later in the season. Iron dynamics were compared to skip-breeding females (females that did not produce a pup during the study year) during the breeding season (October – December, all study years) and molt (January – February, all study years). Females were identified as skip-breeders from consistent surveys of the breeding colonies every 1–3 days^88^ and/or confirmation that skip-breeding females were fully molted by late summer whereas postpartum females were un-molted^89^. In the unlikely event that a female that had a still-birth or that lost a pup due to early neonatal mortality was inadvertently classified as a skip-breeder, these females would still not have undergone the large costs of lactation. Any additional loss of iron associated with giving birth or a few days of suckling in a mis-classified skip-breeder would have slightly diminished the differences between reproductive classes observed in this study, further supporting that there are dramatic differences in physiology between animal reproductive groups and the differences we observed may actually be slightly conservative. This study primarily consisted of prime-age females, with 79% of animals being of known-age and 73% of the study animals were known to be between 10 and 20 years old. A subset of females were handled twice. In 2010–2012, these were skip-breeding females handled during the January – February molt, and the animals returned the following October as skip-breeders (*n* = 8) or gave birth (7 dpp; *n* = 12). From 2013–2017, skip-breeders (*n* = 24) and post-partum females (35 dpp, *n* = 46) were handled in November and recaptured approximately 2 months later in January – February (late-summer molt, or 95 dpp [post-weaning]).

Animals were captured with a hoop net and sedated with Telazol (1.0 mg kg^−1^ tiletamine/zolazepam) or ketamine and midazolam (2.0 and 0.1 mg kg^−1^, respectively) administered intramuscularly (I.M.). Following a 10–15 min induction period, animals were given 0.5 mg kg^−1^ ketamine and 0.025 mg kg^−1^ diazepam or midazolam intravenously (I.V.) as necessary for animals to remain sedated and eupneic^7,90^. Animals were weighed using a sling, tripod, and scale. Body composition (%lipid) and lean body mass was determined for each animal using tritiated water dilution as described in Shero et al. 2014; 2015^17,91^ (Table 1). Blood samples were collected in EDTA (for hematology), heparinized (for ferritin, ceruloplasmin, and blood volume measurements) and serum-separator tube (SST; for iron quantification) vacutainers^TM^ from the extradural vein. Serum/plasma was separated by centrifugation, and all tissue samples collected during this study were stored at –80 °C until laboratory analyses.

### Iron stores and mobilization

Serum iron and iron-binding capacities were measured using a Pointe Scientific, Inc., Iron TIBC kit (I7504). Briefly, serum iron was measured after adding 220 mM hydroxylamine hydrochloride at pH 4.5 to serum to facilitate the release and reduction of transferrin-bound iron. In a separate assay, Unsaturated Iron-Binding Capacity (UIBC) was measured by bringing samples to an alkaline pH (Tris 500 mM, Sodium azide 0.055 w/v, at pH 8.1) and adding a known concentration of ferrous ions that bind with transferrin at the unsaturated binding sites. The difference between the amount of ferrous ion added and the unbound iron measured was taken to be UIBC. For both serum iron and UIBC, concentrations were measured at λ = 560 nm before and after treatment with chromogenic ferrozine and a 10 min incubation at 37 °C. For each individual, Total Iron-Binding Capacity (TIBC) was calculated as the summation of serum iron and UIBC. The percentage of TIBC (an index of transferrin binding sites), occupied with iron (i.e., serum iron concentration) was determined to be the transferrin saturation (Tf-Sat, %). Samples were run in quadruplicate in a Molecular Devices SpectraMax 340 microplate reader (Molecular Devices, Inc., Sunnyvale, CA, USA). Plasma ferritin and ceruloplasmin concentrations were measured at the Kansas State Veterinary Diagnostic Laboratory through enzyme-linked immunoassay (ELISA) and a colorimetric reaction through the oxidative activity of p-phenylenediamine, respectively.

Given that iron is more abundant in the environment than in biological samples, we have stringently followed rigorous cleaning procedures for trace metal analysis to prevent any potential contamination. To determine the amount of iron transferred from female to pup via the milk, 50–60 IU Oxytocin was injected into the mammary gland to facilitate milk let-down, the mammary gland was cleaned with isopropyl alcohol and sterile gauze after the capture net was removed, and milk was manually expressed using a hand pump; all samples were stored in plastic. At the laboratory, all glassware was acid washed prior to use with HCl and HNO_3_ in a sonication bath. Milk was weighed gravimetrically, freeze-dried, and acid digested with repeated additions of 8 N nitric acid and 30% hydrogen peroxide refluxed at 90 °C. Following acid digestion, samples were adjusted to 1% HNO_3_ at 500x dilution, centrifuged, and filtered. Milk iron concentrations were measured with an Agilent 7500c Inductively Coupled Plasma Mass Spectrometer (ICP-MS) with an octopole reaction system (Agilent Technologies, Inc., Santa Clara, CA, USA), alongside Continuing Calibration Verification (recoveries: 103–104%) and NIST standards (NIST 1640e, recovery: 113%), and method blanks were carried throughout the entire sample preparation and analytical processes.

### Hematology and blood volume

To evaluate whether there were shifts in physiology associated with lactation, hematocrit (Hct; packed red blood cell volume) was determined by whole blood centrifugation, and hemoglobin (Hb) concentrations were measured using the cyanomethemoglobin assay with Drabkin’s reagent (Sigma Kit 625 A) and a UV/Vis Beckman series 530 spectrophotometer (Beckman Coulter, Inc., Fullerton, CA, USA) at λ = 540 nm with concentrations of samples calculated through a linear regression of Hb standards (Pointe Scientific, Inc.). Mean corpuscular hemoglobin concentration (MCHC) was calculated as:

1 $${{{{{\rm{MCHC}}}}}}( \% )=({{{{{\rm{Hb}}}}}};{{{{{\rm{g}}}}}}\,{{{{{{\rm{dL}}}}}}}^{-1})/{{{{{\rm{Hct}}}}}}\times 100.$$

Medix^TM^ Ery-Tic RBC test kits were used to count red blood cells (RBCs) (Medix Corp., Newbury Park, CA, USA).

Plasma volume (PV) and blood volume (BV) were determined using the Evan’s Blue dye technique. After a pre-injection blood sample was collected, ~0.5–1.2 mg kg^−1^ Evan’s Blue dye was administered into the extradural vein. Syringes used to distribute the dye were pre-weighed and flushed with blood to accurately determine the amount of dye injected for each animal. Injection was followed by three consecutive blood draws ~10 mins apart, with the exact time of sample collections recorded. After centrifugation, the plasma background absorbance values were measured at λ = 740 nm and were subtracted from optical density at λ = 624 nm. Evan’s Blue dye stock (40 mg mL^−1^) was used to construct standard curves and determine sample concentrations. Dilution of the dye was used to calculate PV as described in Foldager & Blomqvist (1991)^92^ and El-Sayed et al. (1995)^93^ and BV was calculated as:

2 $${{{{{\rm{Blood}}}}}}\,{{{{{\rm{Volume}}}}}}({{{{{\rm{L}}}}}})=\frac{{{{{{\rm{Plasma}}}}}}\,{{{{{\rm{Volume}}}}}}({{{{{\rm{L}}}}}})}{(100-{{{{{\rm{Hct}}}}}})/100}.$$

### Muscle myoglobin and enzyme kinetics

A skeletal muscle biopsy from the *Longissimus dorsi* (*LD*) swimming muscle was collected with a 6 mm biopsy punch or cannula and was immediately frozen in liquid nitrogen or dry ice before being stored at –80 °C until analysis. Muscle Mb concentrations (mg g wet tissue^−1^) were assayed following Reynafarje 1963^94^ as modified for microwell format by Prewitt et al. 2010^95^ and analyzed in quadruplicate alongside a lyophilized myoglobin horse standard (Sigma Aldrich) and previously assayed harbor seal (*Phoca vitulina*) and Weddell seal tissue (values were only accepted if intra-assay CVs were < 10%; Inter-assay CVs: 8.2%). Repeated samples from the same individual, collected at different timepoints across the austral summer were preferentially run on the same plate to reduce inter-assay variability in assessing longitudinal changes in Mb. Samples were read at both λ = 538 and 568 nm to account for any Hb contamination.

To evaluate whether shifts in iron metabolism have downstream effects on aerobic and anaerobic ATP production potential, citrate synthase (CS), *β*-hydroxyacyl CoA dehydrogenase (HOAD), and lactate dehydrogenase (LDH) kinetic activities (IU g^−1^ wet mass muscle) were measured. Spectrophotometric assays were run according to the procedures described by Polasek et al. 2006^96^ and Prewitt et al. 2010^95^ under substrate saturating conditions held at 37 °C. A buffer blank and a previously assayed muscle sample of known activity were measured as controls along with all experimental samples analyzed in quadruplicate (Inter-assay CVs: CS 12.7%, HOAD 12.9%, LDH 7.9%). CS:HOAD ratios were evaluated to determine the relative amount of aerobic metabolism that utilizes *β*-oxidation of fatty acids. Values less than 1 indicate higher dependence on lipid stores. The LDH:CS ratios were calculated to compare potentials for aerobic versus anaerobic metabolism^96^.

### Total iron, body oxygen stores, and the cADL

Total circulating and muscle iron stores were calculated as the summation of:

3 $${{{{{\rm{Circulating}}}}}}\,{{{{{\rm{iron}}}}}}\,{{{{{\rm{stores}}}}}}({{{{{\rm{g}}}}}})={{{{{\rm{Hb}}}}}}({{{{{\rm{g}}}}}}\,{{{{{{\rm{dL}}}}}}}^{-1})\times 10\times {{{{{\rm{BV}}}}}}({{{{{\rm{Liters}}}}}})\times 0.00347\,{{{{{\rm{g}}}}}}\,{{{{{\rm{Fe}}}}}}$$

and

4 $${{{{{\rm{Muscle}}}}}}\,{{{{{\rm{iron}}}}}}\,{{{{{\rm{stores}}}}}}({{{{{\rm{g}}}}}})={{{{{\rm{Mb}}}}}}({{{{{\rm{mg}}}}}}\,{{{{{\rm{g}}}}}}\,{{{{{{\rm{tissue}}}}}}}^{-1})\times ({{{{{\rm{Muscle}}}}}}\,{{{{{\rm{mass}}}}}},{{{{{\rm{kg}}}}}})\times 0.00321\,{{{{{\rm{g}}}}}}\,{{{{{\rm{Fe}}}}}}$$

where total muscle mass was estimated as 38% of lean body mass^12^. The amount of iron per gram of hemoprotein was determined as the molecular mass of Fe divided by the molecular mass of Hb/Mb (where Hb has 4 Fe atoms; Mb contains 1).

To then calculate total body oxygen (TBO_2_) stores, blood O_2_ stores were first calculated from Hb, Hct, and BV in arterial and venous systems with the following assumptions: (1) Hb has an O_2_ carrying capacity of 1.34 mL O_2_ g^−1^ [Hb], (2) arterial blood is 33% of total BV, with the remaining 66% blood in the venous system, (3) the maximum O_2_ saturation possible in the arterial system is 95%, and a minimum saturation of 20% after O_2_ has been transported to other tissues, and (4) venous blood is assumed to have 5% less volume than the starting arterial O_2_ stores and can be extracted to zero^12,97,98^. Muscle O_2_ stores were determined for each animal assuming that Mb also had an O_2_ carrying capacity of 1.34 mL O_2_ g^−1^ [Mb] and total muscle mass was 38% of lean body mass (as above). Lung O_2_ stores were calculated as follows:

5 $${{{{{\rm{Lung}}}}}}\;\,{{{{{{\rm{O}}}}}}}_{2}=Vi\;\times \;0.15{{{{{{\rm{FO}}}}}}}_{2}.$$

*V*_*i*_ is the estimated diving lung volume (in liters) calculated as 0.5 × 0.10(total body mass)^0.96^ which assumes that lung volume is at 50% total capacity at the onset of diving, and 0.15 FO_2_ is the partial pressure of O_2_ in the lungs^13^. Blood, muscle, and lung O_2_ stores were summed to give TBO_2_ stores of animals for which all measurements were possible^12,97,99^. Each animal’s diving metabolic rate (DMR) was estimated from 1.6 × Kleiber^66,100^. Calculated aerobic dive limits (cADLs) were determined by dividing TBO_2_ stores (in mL O_2_) by DMR (mL O_2_ min^−1^), and TBO_2_ stores were scaled to lean body mass to control for dramatic seasonal and lactation-associated changes in lipid stores^17^, prior to making comparisons among reproductive classes.

### Links with dive behavior

To determine whether changes in hemoproteins do in fact translate to changes in dive behavior and foraging abilities, the same Weddell seals for which physiologic measurements were collected in this study were also instrumented to track dive durations. As described previously in Shero et al.^31^, Sea Mammal Research Unit (SMRU) Conductivity Temperature Depth-Satellite Relay Dive Loggers (CRD-SRDLs) weighing 600 g were deployed to measure overwinter (gestational) foraging behaviors from 2010–2012. Instruments were attached to the fur on 20 adult female Weddell seals’ heads using 5 min epoxy (Loctite® or Devcon®). Data were transmitted as compressed dives to the Collecte Localisation Satellites, Advanced Research and Global Observation Satellite System (CLS ARGOS). All females were skip-breeders at the time of tagging in January-February, and 12 females returned the following year and successfully gave birth while 8 females failed to produce a pup the following year (i.e., returned as skip-breeders). Skip-breeder behavioral data transmitted into the summer period coinciding with lactation (November), and post-partum female dive durations during mid-lactation from Sato et al. 2003^101^ were used in this study for comparison between reproductive classes.

From 2013–2017, 59 animals received LOTEK LAT1800 archival time depth recorders (TDRs) to collect summer foraging behavioral data as described in Beltran et al. 2021^30^. TDRs were deployed on the hind flippers, in the interdigital webbing and instruments were programmed to collect behavioral information at 6 s intervals. Nineteen of these seals were skip-breeders when tags were deployed (November-December), and 40 were post-partum females (instruments deployed at 35 dpp). TDRs had to be physically recovered (in January-February; ~95 dpp) to obtain the archived data. Skip-breeders and post-partum females were initially tagged and instruments recovered in the same locations (i.e., skip-breeders were tagged at sites with the same bathymetric features as post-partum seals). All animals that received dive instruments from 2010–2017 were also outfitted with a VHF transmitter (LOTEK or Advanced Telemetry Systems) to facilitate recapture.

### Statistical analysis

Linear mixed effect models (LME; in ‘lme4’ and ‘lmerTest’ packages) were used to test for differences in iron mobilization and physiologic dive capacities among reproductive classes (included as cofactors), and animal ID was included as a random effect^102^. Post-hoc comparisons were made using Bonferroni corrections (“multcomp” package). Linear regressions were used to determine whether iron stores from different tissues were correlated, and the relationship between iron status at different time points throughout the study (i.e., during lactation and the subsequent post-weaning period). Generalized additive mixed effect models (GAMMs; in ‘gamm4’ package) were used to test for differences in dive duration among reproductive classes, with animal ID included as a random effect. Models were examined to ensure homoscedasticity and that there was not overdispersion of residuals. Outliers were identified using Cook’s distance plots generated from model fit and removed. All analyses were performed in R (v. 4.1.0) and significance was set at the α = 0.05 level; all results are reported as mean ± standard error.

### Reporting summary

Further information on research design is available in the Nature Research Reporting Summary linked to this article.

## Supplementary information

Supplementary Information

Reporting Summary

## Data Availability

All data generated in this study have been deposited and made publicly available through the Antarctic Master Directory, U.S. Antarctic Program (USAP) Data Center^103^. This repository contains all raw and processed data, and source data for comparative figures. Files are provided through the permanent link: https://www.usap-dc.org/view/dataset/601575.
