# Supplementary material for: Iron mobilization during lactation reduces oxygen stores in a diving mammal
Source: Nat Commun. 2022 Aug 2;13:4322. doi: 10.1038/s41467-022-31863-7 (PMC9345918; doi:10.1038/s41467-022-31863-7)
Supplement: Supplementary file 1 — Supplementary Information [file 41467_2022_31863_MOESM1_ESM.pdf]

Iron mobilization during lactation reduces oxygen stores in a diving mammal

Michelle R. Shero[1*], Amy L. Kirkham[2,3], Daniel P. Costa[4], Jennifer M. Burns[5]

[1]Biology Department, Woods Hole Oceanographic Institution, 266 Woods Hole Rd, Woods Hole, MA 02543
[2]Department of Biological Sciences, University of Alaska Anchorage, 3101 Science Circle, Anchorage, AK 99508
[3]College of Fisheries and Ocean Sciences, University of Alaska Fairbanks, 17101 Point Lena Loop Road, Juneau, AK 99801, USA
[4]Institute of Marine Science, Ecology and Evolutionary Biology, University of California Santa Cruz, CA 95060
[5]Department of Biological Sciences, Texas Tech University, Lubbock, TX 79409, USA

Supplementary Tables

Supplementary Table 1. Shifts in Weddell seal oxygen stores and diving metabolic rate (DMR) associated with lactation. Mean ± SE Weddell seal blood, muscle, lung (and summed total) O_2 stores in skip-breeding and post-partum females across the austral summer lactation and post-weaning periods. Calculated DMRs were used to calculate the aerobic dive limit. Statistical significance was detected using Linear Mixed-Effect models and post-hoc comparisons with Bonferroni correction. Sample size for each measure is shown in parentheses and different letters indicate significant differences in physiological parameter between reproductive classes.

	**Skip-Breeders**		**Post-Partum Females**			**Effect of Reproductive Class**
	**Early-Summer (Lactation Period)**	**Late-Summer**	**Early-Summer Beginning Lactation (7 dpp)**	**Early-Summer End Lactation (35 dpp)**	**Late-Summer Post-weaning (95 dpp)**	**LME F-statistic; P-value**
Blood O_2 (L)	16.0 ± 0.6^{a} *(60)*	14.8 ± 0.4^{b} *(82)*	15.4 ± 0.8^{ab} *(16)*	13.6 ± 0.3^{b} *(63)*	12.8 ± 0.3^{b} *(56)*	$\mathbf{F_{4,142} = 7.2, P < 0.001}$
Muscle O_2 (L)	10.4 ± 0.5^{ab} *(45)*	10.1 ± 0.3^{a} *(67)*	13.0 ± 0.5^{b} *(12)*	8.6 ± 0.3^{c} *(45)*	8.7 ± 0.3^{c} *(36)*	$\mathbf{F_{4,101} = 8.1, P < 0.001}$
Lung O_2 (L)	2.30 ± 0.06^{a} *(60)*	2.03 ± 0.04^{b} *(84)*	2.44 ± 0.08^{a} *(16)*	1.69 ± 0.03^{c} *(63)*	1.86 ± 0.03^{b} *(59)*	$\mathbf{F_{4,113} = 46.3, P < 0.001}$
Total Body O_2 (L)	28.6 ± 1.1^{a} *(45)*	26.8 ± 0.6^{ab} *(66)*	30.3 ± 1.0^{ab} *(12)*	24.1 ± 0.6^{bc} *(45)*	23.0 ± 0.5^{c} *(36)*	$\mathbf{F_{4,87} = 7.0, P < 0.001}$
DMR (L O_2 min^{-1})	1.58 ± 0.03^{a} *(60)*	1.43 ± 0.02^{b} *(84)*	1.65 ± 0.04^{a} *(16)*	1.24 ± 0.02^{c} *(63)*	1.34 ± 0.02^{b} *(59)*	$\mathbf{F_{4,113} = 45.4, P < 0.001}$
DMR (mL O_2 kg lean $mass^{-1}$)	6.53 ± 0.07^{a} *(56)*	6.21 ± 0.04^{b} *(82)*	6.29 ± 0.10^{ab} *(15)*	6.49 ± 0.05^{a} *(62)*	5.77 ± 0.04^{c} *(59)*	$\mathbf{F_{4,143} = 52.1, P < 0.001}$

Supplementary Figures

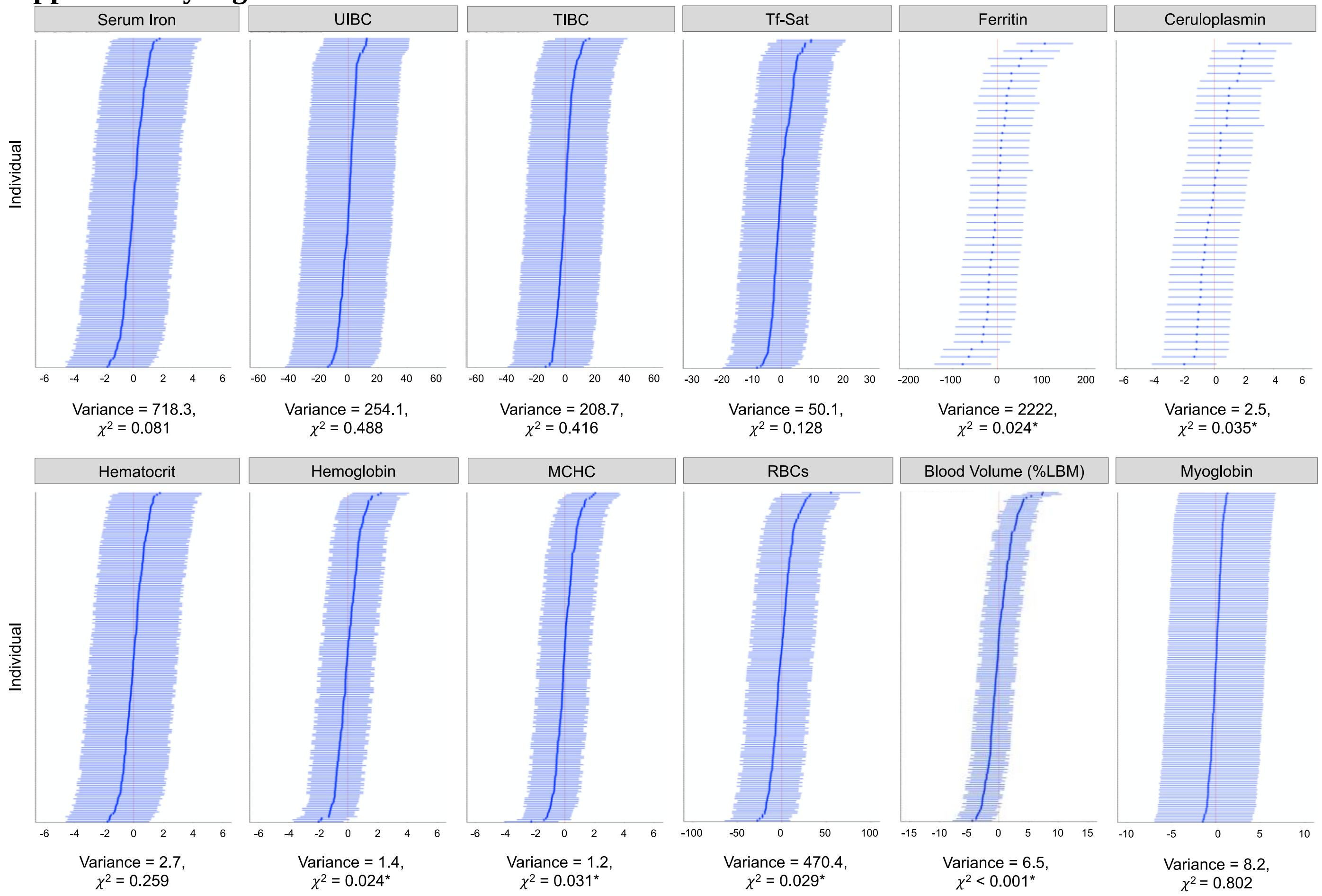

Supplementary Figure 1. Individual-effects on Weddell seal physiological parameters. Variance associated with individual animals (i.e., random effects) that contributed to linear mixed effect models comparing hematology, hemoproteins, and serum biochemistry between reproductive classes. Error bars denote 95% confidence intervals. *n* for Serum iron: 277; Unsaturated Iron-Binding Capacity (UIBC): 277; Total Iron-Binding Capacity (TIBC): 277; Transferrin Saturation (Tf-Sat): 277; Ferritin: 86; Ceruloplasmin: 86; Hematocrit: 289; Hemoglobin: 288; Mean Corpuscular Hemoglobin Concentration (MCHC): 285; Red blood cells (RBCs): 261; Blood Volume as a percentage of lean body mass (LBM): 268; Myoglobin: 214.

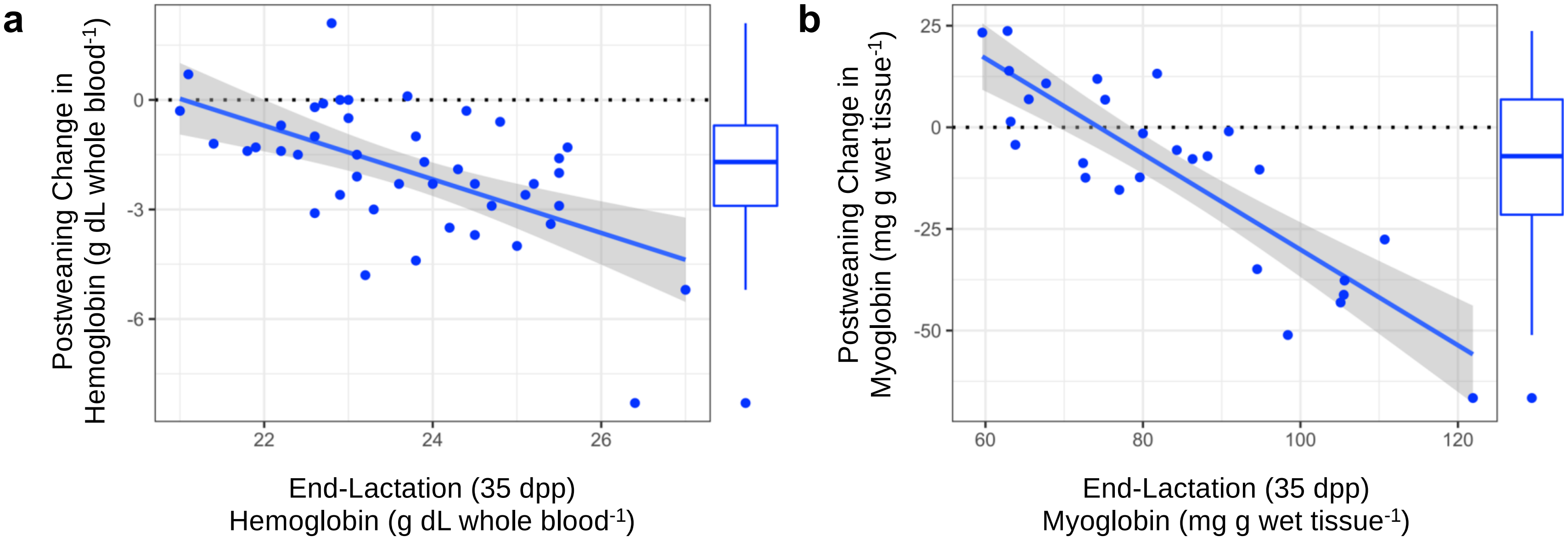

Supplementary Figure 2. Hemoprotein concentration at the end of lactation determined the magnitude of hemoprotein loss post-weaning, on an absolute-basis. Female Weddell seals with higher (**a**) blood hemoglobin and (**b**) muscle myoglobin at the end of lactation (35 dpp) exhibited greater declines in hemoprotein concentrations post-weaning (by 95 dpp) on an absolute basis. Grey bands depict the 95% confidence interval around the regression model line (center of error bands); boxplots depict the distribution of changes in hemoprotein concentrations where boxes encompass the interquartile range, the center line denotes the median, whiskers encompass range of values, and points above or below the whiskers are greater/less than 1.5-times the interquartile range. *n* for Hb = 45; Mb = 27.
