# Supplementary material for: Iron mobilization during lactation reduces oxygen stores in a diving mammal
Source: Nat Commun. 2022 Aug 2;13:4322. doi: 10.1038/s41467-022-31863-7 (PMC9345918; doi:10.1038/s41467-022-31863-7)
Supplement: Supplementary file 2 — Reporting Summary [file 41467_2022_31863_MOESM2_ESM.pdf]

# nature portfolio

Corresponding author(s): Michelle Shero

Last updated by author(s): Jun 14, 2022

# Reporting Summary

Nature Portfolio wishes to improve the reproducibility of the work that we publish. This form provides structure for consistency and transparency in reporting. For further information on Nature Portfolio policies, see our Editorial Policies and the Editorial Policy Checklist.

## Statistics

For all statistical analyses, confirm that the following items are present in the figure legend, table legend, main text, or Methods section.

| n/a | Confirmed | |
|---|---|---|
| ☐ | ☒ | The exact sample size (*n*) for each experimental group/condition, given as a discrete number and unit of measurement |
| ☐ | ☒ | A statement on whether measurements were taken from distinct samples or whether the same sample was measured repeatedly |
| ☐ | ☒ | The statistical test(s) used AND whether they are one- or two-sided<br>*Only common tests should be described solely by name; describe more complex techniques in the Methods section.* |
| ☐ | ☒ | A description of all covariates tested |
| ☐ | ☒ | A description of any assumptions or corrections, such as tests of normality and adjustment for multiple comparisons |
| ☐ | ☒ | A full description of the statistical parameters including central tendency (e.g. means) or other basic estimates (e.g. regression coefficient) AND variation (e.g. standard deviation) or associated estimates of uncertainty (e.g. confidence intervals) |
| ☐ | ☒ | For null hypothesis testing, the test statistic (e.g. *F*, *t*, *r*) with confidence intervals, effect sizes, degrees of freedom and *P* value noted<br>*Give P values as exact values whenever suitable.* |
| ☒ | ☐ | For Bayesian analysis, information on the choice of priors and Markov chain Monte Carlo settings |
| ☒ | ☐ | For hierarchical and complex designs, identification of the appropriate level for tests and full reporting of outcomes |
| ☒ | ☐ | Estimates of effect sizes (e.g. Cohen's *d*, Pearson's *r*), indicating how they were calculated |

*Our web collection on statistics for biologists contains articles on many of the points above.*

## Software and code

Policy information about availability of computer code

| | |
|---|---|
| Data collection | No software was used to generate the data. |
| Data analysis | Statistical analyses were conducted in R (v. 4.1.0). Linear mixed effect models were conducted with the 'lme4' and 'lmerTest' packages, and post-hoc comparisons were made using the 'multcomp' package. Generalized additive mixed models were conducted using 'gamm4'. |

For manuscripts utilizing custom algorithms or software that are central to the research but not yet described in published literature, software must be made available to editors and reviewers. We strongly encourage code deposition in a community repository (e.g. GitHub). See the Nature Portfolio guidelines for submitting code & software for further information.

## Data

Policy information about availability of data

All manuscripts must include a data availability statement. This statement should provide the following information, where applicable:

- Accession codes, unique identifiers, or web links for publicly available datasets
- A description of any restrictions on data availability
- For clinical datasets or third party data, please ensure that the statement adheres to our policy

All data generated in this study have been deposited and made publicly available through the Antarctic Master Directory, U.S. Antarctic Program (USAP) Data Center. Files are provided through the permanent link: https://www.usap-dc.org/view/dataset/601575.
Full citation:

Shero, M. Weddell seal iron dynamics and oxygen stores across lactation. U.S. Antarctic Program (USAP) Data Center. doi: https://doi.org/10.15784/601575. (2022).

## Human research participants

Policy information about studies involving human research participants and Sex and Gender in Research.

| | |
|---|---|
| Reporting on sex and gender | *Use the terms sex (biological attribute) and gender (shaped by social and cultural circumstances) carefully in order to avoid confusing both terms. Indicate if findings apply to only one sex or gender; describe whether sex and gender were considered in study design whether sex and/or gender was determined based on self-reporting or assigned and methods used. Provide in the source data disaggregated sex and gender data where this information has been collected, and consent has been obtained for sharing of individual-level data; provide overall numbers in this Reporting Summary. Please state if this information has not been collected. Report sex- and gender-based analyses where performed, justify reasons for lack of sex- and gender-based analysis.* |
| Population characteristics | *Describe the covariate-relevant population characteristics of the human research participants (e.g. age, genotypic information, past and current diagnosis and treatment categories). If you filled out the behavioural & social sciences study design questions and have nothing to add here, write "See above."* |
| Recruitment | *Describe how participants were recruited. Outline any potential self-selection bias or other biases that may be present and how these are likely to impact results.* |
| Ethics oversight | *Identify the organization(s) that approved the study protocol.* |

Note that full information on the approval of the study protocol must also be provided in the manuscript.

# Field-specific reporting

Please select the one below that is the best fit for your research. If you are not sure, read the appropriate sections before making your selection.

☐ Life sciences ☐ Behavioural & social sciences ☒ Ecological, evolutionary & environmental sciences

For a reference copy of the document with all sections, see nature.com/documents/nr-reporting-summary-flat.pdf

# Ecological, evolutionary & environmental sciences study design

All studies must disclose on these points even when the disclosure is negative.

| | |
|---|---|
| Study description | This study tested whether female Weddell seals mobilize and transfer large quantities of iron to their pups during lactation, and if iron offload would draw from the female's own endogenous heme stores and dive capacities. Adult female Weddell seals were handled throughout the austral summer. The post-partum females were our experimental group and the animals were handled at the start of lactation (n=17), end of lactation (n=64), and post-weaning (n=62). To test whether iron mobilization was indeed due to lactation the study control group (skip-breeders that did not have a pup) were handled during the same timeframe during early and late-summer (n=63; 85), These reproductive groups were co-factors in statistical analyses.<br><br>A subset of females were handled twice. In 2010-2012, these were skip-breeding females handled during the January-February molt, and the animals returned the following October as skip-breeders (n = 8) or gave birth (7 days post-partum, dpp; n = 12). From 2013-2017, skip-breeders (n = 24) and post-partum (35 dpp, n = 46) were handled in November and recaptured approximately 2 months later in January-February (late-summer molt, or 95 dpp [post-weaning]). These are detailed in the Methods secion and Tables, and statistical analyses included animal ID as a random effect to account for repeated measures. |
| Research sample | Cohorts of two different reproductive classes (skip-breeder; post-partum) adult female Weddell seals were handled across the austral summer. These two cohorts were chosen to test for the impact of lactation on iron mobilization and hemoprotein maintenance. Post-partum femalse were handled at the beginning and end of lactation to track iron dynamics across lactation, and after weaning their pups because hemoprotein production can exhibit a time lag. Skip-breeders were handled at equivalent timepoints throughout the austral summer. Skip-breeders were handled during early summer, when post-partum females give birth) and late summer (when post-partum females had weaned their pups).<br><br>Females were not captured until a few days after parturition to ensure sufficient bonding with the pup prior to handling. Similarly, a standardized timepoint towards the end of lactation was chosen because some females can forage intensively late in lactation, making them difficult to capture just prior to weaning. Animals were all either previously flipper-tagged with a unique identification number, or tagged at handling, to determine birth dates and facilitate recapture later in the season. Iron dynamics were compared to skip-breeding females (females that did not produce a pup during the study year) during the breeding season (October – December, all study years) and molt (January – February, all study years). Females were identified as skip-breeders from consistent surveys of the breeding colonies every 1-3 days and/or confirmation that skip-breeding females were fully molted by late summer whereas postpartum females were un-molted. In the unlikely event that a female that had a still-birth or that lost a pup due to early neonatal mortality was inadvertently classified as a skip-breeder, these females would still not have undergone the large costs of lactation. Any additional loss of iron associated with giving birth or a few days of suckling in a mis-classified skip-breeder would have slightly diminished the differences between reproductive classes observed in this study, further supporting that there are dramatic differences in physiology between animal reproductive groups and the differences we observed may actually be slightly conservative. |

| | |
|---|---|
| | This study primarily consisted of prime-age females, with 79% of animals being of known-age and 73% of the study animals were known to be between 10 and 20 years old.<br><br>All study animals were adult female Weddell seals (Leptonychotes weddellii) from the world's southernmost population in Erebus Bay, Antarctica and the Victorialand coastline, Antarctica. Animal reproductive status and timing of sampling was the main criteria in study design development; no animals were experimentally manipulated. |
| Sampling strategy | Animals included in this study were part of two different projects, and sample size was determined using a power analysis to achieve a statistical power of >80%. Specific animals were chosen based on visual observation (that animals were adult females) if not previously tagged. The majority of animals had a unique ID flipper tag and we were able to confirm exact age.<br><br>Animals were captured with a hoop net and sedated with Telazol (1.0 mg kg-1 tiletamine/zolazepam) or ketamine and midazolam (2.0 and 0.1 mg kg-1, respectively) administered intramuscularly (I.M.). Following a 10-15 min induction period, animals were given 0.5 mg kg-1 ketamine and 0.025 mg kg-1 diazepam or midazolam intravenously (I.V.) as necessary for animals to remain sedated and eupneic. Animals were weighed using a sling, tripod, and scale. Body composition was determined using isotopic dilution techniques. Blood was collected from the extradural vein for hematology and serum and plasma collection. Blood volume was measured by dilution of Evans Blue dye in the bloodstream. A muscle biopsy was collected with a 6 mm biopsy punch. Animals were also outfitted with dive recorders. |
| Data collection | Field data collection was conducted by a team of 4-6 people; each animal was handled 1-2 times during the course of the study, as longitudinal measurements provide stronger evidence of shifts in animal physiology but this was balanced against the need to also minimize impacts to the animals. Samples were stored at -80C and shipped back to the United States with appropriate import permits. In the field, everyone from the 4-6 person field team recorded data and it was collated by Jennifer Burns. All the laboratory data for iron and hemoprotein measurements were collected using spectrophotometers and microplate equipment and recorded by the lead author, Michelle Shero. Body composition analyses were conducted using liquid scintillation counters and recorded by Amy Kirkham. |
| Timing and spatial scale | Field sampling occurred from January 2010 to February 2017. Animals were sampled across the austral summer (from October through February), and all samples were collected from Erebus Bay and the Victorialand coastline. These were chosen for the first project included in this study with animals being handled during late summer as this was the best time for dive behavioral recorders to be attached to the animals (after the molt). The second study concluded at the end of February after postpartum females typically return to the study area such that they could be captured for sampling post-weaning. |
| Data exclusions | Exclusion criteria were pre-established and outliers identified based on Cook's distance plots and excluded from analyses. This was to remove outlying points that would have highly influenced models. |
| Reproducibility | While this was not a manipulative experiment that could be independently repeated multiple times, changes in physiology were determined across multiple years in this study. This was necessary to achieve an adequate sample size, and also demonstrates that iron mobilization during lactation leads to a decline in heme stores - in multiple years. In laboratory analyses, care was taken to measure physiology of a 'pooled' or 'control' sample such that inter- and intra-assay coefficient of variations could be calculated and further ensure that measurements were repeatable across assay 'runs'. |
| Randomization | Study animals were allocated into groups based on their age and reproductive status. Age was confirmed by observation (animal size; first date seen with a pup) and demographic records when available. |
| Blinding | Blinding was not possible due to the extent that our field team had to search for animals fitting specific criteria (age, reproductive status) to be included in the study. |

Did the study involve field work? ☒ Yes ☐ No

## Field work, collection and transport

| | |
|---|---|
| Field conditions | Sampling occurred in relatively benign weather conditions (> -20C temperature) to ensure our team could handle the animals safely. |
| Location | All sampling occurred in Erebus Bay (~77-degrees S, 165-degrees E) and the Victorialand coastline (76-degrees S, 162-degrees E), in the Ross Sea, Antarctica. |
| Access & import/export | Access to habitats was granted under the Antarctic Conservation Act permits, and animal handling procedures were approved by institutional IACUCs and NMFS MMPA permits: 17411-03 (issued: June 11, 2013) and 87-1851-04 (issued: January 29, 2007). NMFS permits also allowed sample import into the United States. |
| Disturbance | We took great lengths and employed best practices to minimize the impacts of animal handlings. Any stress was minimized through sedation and local analgesics, and having sufficient trained personnel in the field. Only animals in good condition were handled (i.e., we did not sedate animals with large visible wounds, or that were emaciated). A veterinarian or trained personnel was put in charge of the animal sedations. Behavioral data collected from these animals suggests any disturbance was very short in duration, and animals resumed normal dive activities within a few hours after release. |

# Reporting for specific materials, systems and methods

We require information from authors about some types of materials, experimental systems and methods used in many studies. Here, indicate whether each material, system or method listed is relevant to your study. If you are not sure if a list item applies to your research, read the appropriate section before selecting a response.

## Materials & experimental systems

| n/a | Involved in the study |
|---|---|
| x | Antibodies |
| x | Eukaryotic cell lines |
| x | Palaeontology and archaeology |
|  | x Animals and other organisms |
| x | Clinical data |
| x | Dual use research of concern |

## Methods

| n/a | Involved in the study |
|---|---|
| x | ChIP-seq |
| x | Flow cytometry |
| x | MRI-based neuroimaging |

## Animals and other research organisms

Policy information about studies involving animals; ARRIVE guidelines recommended for reporting animal research, and Sex and Gender in Research

| | |
|---|---|
| Laboratory animals | This study did not involve laboratory animals. |
| Wild animals | Adult female Weddell seals were captured via hoop-net and sedated. This study primarily consisted of prime-age females, with 79% of animals being of known-age and 73% of the study animals were known to be between 10 and 20 years old, verified by demographic records. Unknown age animals were still adults based on observation and body size. After collecting our physiological samples, all animals were allowed to recover from sedation and were released back into the wild. |
| Reporting on sex | Findings apply only to female Weddell seals, because the focus of the study was on lactational effects of iron transfer. Males are not included in this study. |
| Field-collected samples | Animals were all free-living Weddell seals and therefore ambient conditions were not controlled. |
| Ethics oversight | All work in this study complies with ethical regulations, and animal handling protocols were approved by the University of Alaska Anchorage and Fairbanks, and University of California Santa Cruz's IACUC committees. Research and sample import to the United States were authorized under NMFS MMPA permits: 87-1851-04 and 17411-03. Research activities were also approved through Antarctic Conservation Act permits while at McMurdo Station. |

Note that full information on the approval of the study protocol must also be provided in the manuscript.
